# Phylogenetic analysis of endogenous viral elements in the rice genome reveals local chromosomal evolution in *Oryza* AA-genome species

**DOI:** 10.3389/fpls.2023.1261705

**Published:** 2023-10-27

**Authors:** Nozomi Saito, Sunlu Chen, Katsuya Kitajima, Zhitong Zhou, Yohei Koide, Jaymee R. Encabo, Maria Genaleen Q. Diaz, Il-Ryong Choi, Kanako O. Koyanagi, Yuji Kishima

**Affiliations:** ^1^ Research Faculty of Agriculture, Hokkaido University, Sapporo, Japan; ^2^ State Key Laboratory of Crop Genetics and Germplasm Enhancement, Jiangsu Collaborative Innovation Center for Modern Crop Production, Jiangsu Province Engineering Research Center of Seed Industry Science and Technology, Cyrus Tang Innovation Center for Seed Industry, Nanjing Agricultural University, Nanjing, China; ^3^ Graduate School of Information Science and Technology, Hokkaido University, Sapporo, Hokkaido, Japan; ^4^ Institute of Biological Sciences, College of Arts and Sciences, University of the Philippines, Los Baños, Laguna, Philippines; ^5^ Rice Breeding Platform, International Rice Research Institute, Los Baños, Laguna, Philippines; ^6^ Faculty of Information Science and Technology, Hokkaido University, Sapporo, Hokkaido, Japan

**Keywords:** eRTBVL-D, local chromosomal evolution, *Oryza* AA-genome species, phylogenetic incongruence, rearrangement, introgression

## Abstract

**Introduction:**

Rice genomes contain endogenous viral elements homologous to rice tungro bacilliform virus (RTBV) from the pararetrovirus family *Caulimoviridae*. These viral elements, known as endogenous RTBV-like sequences (eRTBVLs), comprise five subfamilies, eRTBVL-A, -B, -C, -D, and -X. Four subfamilies (A, B, C, and X) are present to a limited degree in the genomes of the Asian cultivated rice *Oryza sativa* (spp. *japonica* and *indica*) and the closely related wild species *Oryza rufipogon*.

**Methods:**

The eRTBVL-D sequences are widely distributed within these and other *Oryza* AA-genome species. Fifteen eRTBVL-D segments identified in the *japonica* (Nipponbare) genome occur mostly at orthologous chromosomal positions in other AA-genome species. The eRTBVL-D sequences were inserted into the genomes just before speciation of the AA-genome species.

**Results and discussion:**

Ten eRTBVL-D segments are located at six loci, which were used for our evolutionary analyses during the speciation of the AA-genome species. The degree of genetic differentiation varied among the eRTBVL-D segments. Of the six loci, three showed phylogenetic trees consistent with the standard speciation pattern (SSP) of the AA-genome species (Type A), and the other three represented phylogenies different from the SSP (Type B). The atypical phylogenetic trees for the Type B loci revealed chromosome region–specific evolution among the AA-genome species that is associated with phylogenetic incongruences: complex genome rearrangements between eRTBVL-D segments, an introgression between the distant species, and low genetic diversity of a shared eRTBVL-D segment. Using eRTBVL-D as an indicator, this study revealed the phylogenetic incongruence of local chromosomal regions with different topologies that developed during speciation.

## Introduction

Endogenous virus elements (EVEs) have been identified in many plant species following sequencing of their genomes (Staginnus and Richert-Poggeler, 2006; Chu et al., 2014). These EVEs originated from once-active viruses that infected a plant cell and incorporated part or all of its genome into the host genome (Staginnus and Richert-Poggeler, 2006). Subsequently, only a few of these EVEs were transmitted from host gametes to offspring through germ cells and passed on to progeny genomes (Feschotte and Gilbert, 2012). Many plant species contain fragmented viral sequences scattered throughout the genome that have lost their function as infectious viruses (Harper et al., 2002). Some, however, have nearly intact virus-like sequences (e.g., from Petunia vein virus) in their genomes and produce viral particles in their cells, though rarely (Richert-Poggeler et al., 2003; Staginnus and Richert-Poggeler, 2006). Most of the endogenous viruses found in plant genomes originated from viruses belonging to the *Caulimoviridae*, a family of pararetroviruses (Harper et al., 1999; Jakowitsch et al., 1999; Ndowora et al., 1999; Chabannes and Caruana, 2013). Pararetroviruses are double-stranded DNA viruses that have reverse transcriptase activity but, because they do not encode integrase, cannot be autonomously inserted into the host genome as retroviruses can (Hull et al., 2000; Peterson-Burch et al., 2000; Harper et al., 2002). Instead, EVEs are inserted into the host genome at the site of a double-stranded break in a chromosome. Liu et al. (2012) reported that EVEs in rice genomes were predominantly within AT-rich sequences comprising the matrix attachment region that formed a loop structure and attached to the nuclear matrix. Structural analysis of EVE sequences showed that many EVEs cannot move through the genome autonomously and, once incorporated into the host genome, remain fixed in place (Hull et al., 2000; Harper et al., 2002). The polymorphisms caused by the insertion of EVEs are evolutionary indicators of a time when certain viruses were actively infectious.

Genome analysis enables a detailed comparison of chromosomal sequences from different species, and phylogenetic analysis using these sequences has led to a significant increase in the characterization of the comparative evolution of many plant species (Zimmer and Wen, 2012; Zimmer and Wen, 2015). As a result, the process of speciation is being elucidated from the relationships among plant species (Nagel et al., 2021). Although significant recent progress has been made both in deciphering the phylogeny of entire plant genomes and in understanding the mechanisms of speciation (Brozynska et al., 2017; Stein et al., 2018), much less is known about the origin and diversification of specific regions of chromosomes, and important questions remain to be answered. For example, have changes in specific chromosomal regions been synchronized with genome-wide evolution, or have they evolved in a manner specific to a particular chromosome region? Indeed, although genes with adaptive functions in evolutionary processes have been analyzed in depth (Strasburg et al., 2012; Bamba et al., 2019), the study of associations between nongenic regions and speciation has been limited to date.

Endogenous RTBV-like sequences (eRTBVLs) are EVEs found in the rice genome that are homologous to rice tungro bacilliform virus (RTBV) (Kunii et al., 2004; Liu et al., 2012). RTBV, a pararetrovirus in the *Caulimoviridae* virus family, is one of the causes of tungro disease of rice, which results in dwarfism, leaf yellowing, and reduced fertility in plants co-infected with rice tungro spherical virus (RTSV) (Hull, 1996). Based on sequence homology, eRTBVLs have been divided into five subfamilies (eRTBVL-A, -B, -C, -D, and -X) (Chen et al., 2014; Chen and Kishima, 2016). Of these five subfamilies, eRTBVL-A, -B, -C, and -X are distributed in the genomes of current Asian rice cultivars and their ancestral wild species, with about 100 copies per genome (Chen et al., 2014). Therefore, it has been suggested that these four eRTBVL subfamilies infected and became inserted into the host genomes during the differentiation of wild and cultivated rice species in Asia (Chen and Kishima, 2016; Chen et al., 2018). By contrast, eRTBVL-D has been found in the genomes of all AA-genome species of the genus *Oryza*, and the insertions are located primarily at orthologous positions among the AA-genome species, whereas no eRTBVL-D has been found in the other *Oryza* species (Chen et al., 2018). These observations suggest that eRTBVL-D infected and became integrated into an ancestral species just before the differentiation of the AA-genome species. In the Nipponbare genome, 15 segments of eRTBVL-D could be identified (Chen et al., 2018). In four of the eRTBVL-D loci, two or three segments are close together, so we designated the positions of eRTBVL-D on the chromosomes as 10 loci that were mostly in orthologous chromosomal locations in the AA-genome species. Most eRTBVL-D sequences were vertically transmitted during speciation in the AA-genome species, but in a few species, some loci are missing, rearranged, and/or translocated.

Sequencing and annotation of AA-genome species has provided comprehensive knowledge regarding their speciation (Zhu et al., 2014; Stein et al., 2018), and these results have clarified the overall evolutionary development of *Oryza* species. However, it is unclear whether the evolution of local chromosome sites reflects the evolution of the entire genome. Therefore, we analyzed the origins of specific regional chromosomal segments and determined when they first arose. eRTBVL-D is an exclusive chromosome constituent whose origin and timing of insertion are clear (Chen et al., 2014). It is of considerable interest to determine how the eRTBVL-D segments with the same origin were inserted into the different chromosomal positions during speciation. Detailed comparisons of orthologous eRTBVL-D segments in the various AA-genome species reveal the different degrees of divergence in a chromosome region–specific manner. Furthermore, eRTBVL-D also enables comparison of local integration sites between species to reveal local structural changes in specific chromosomes. Here, our phylogenetic analyses of eRTBVL-D elements provide evidence both for a recent introgression or a genome exchange between *O. sativa* ssp. *japonica* and *Oryza longistaminata* and for the translocation of a chromosomal segment containing an eRTBVL-D segment during AA-genome speciation.

## Materials and methods

### RTBV and eRTBVL sequences

The RTBV sequence (NCBI accession: NC_001914.1) was used as an outgroup for several analyses. For eRTBVL families, the consensus sequences of eRTBVL-A, -B, and -C were obtained from NCBI (NCBI accessions BR000029.1, BR000030.1, and BR000031.1, respectively) and the consensus sequence of eRTBVL-X used in this study was as described by Chen et al. (2018). According to Chen et al. (2018), collections of eRTBVL-D were employed with a BLASTn search of the *O. sativa japonica* genome using the consensus sequences of six other eRTBVL groups as queries. The segments with the highly reliable hits (*e*-values < 1 × 10^−3^ and lengths > 100 bp) and showing <85% sequence identities to non-eRTBVL-D consensus sequences were collected as eRTBVL-D”.

### Collection of genomic data from *Oryza* species

The eRTBVL-D loci mapped to the Nipponbare genome (*O. sativa* ssp. *japonica*) were used as the references. The genome sequences are available for the seven AA-genome species *O. sativa* ssp. *japonica* and *indica*, *O. rufipogon*, *O. glaberrima*, *O. barthii*, *O. glumaepatula*, *O. meridionalis*, and *O. longistaminata* (**Supplementary Table 1**). These genome sequences were mainly obtained through Gramene (http://gramene.org/) and NCBI (https://www.ncbi.nlm.nih.gov/Traces/wgs/?view=wgs) except for indica (V2plus: https://ars.els-cdn.com/content/image/1-s2.0-S1674205217300424-mmc9.zip) (Zhang et al., 2016). The genome sequence of *O. punctata*, which is an *Oryza* BB-genome species, was also used to examine whether eRTBVL-D sequences are present in the databases of Gramene and NCBI (**Supplementary Table 1**). The BB genome species are considered the closest group genetically to the AA genomic species, with *O. punctata* being the BB genome species, and *O. punctata* is the plant species whose genome has been analyzed the most among the BB genome species (Kim et al., 2007). We selected the candidates for eRTBVL-D orthologous sequences in each genome of the AA-genome species when the corresponding eRTBVL-D in the Nipponbare genome possessed identical contiguous flanking sequences. The selected candidate sequences were confirmed with a highly reliable hit (*e*-values < 1 × 10^-3^ and lengths > 100 bp) with ones in the Nipponbare genome and were judged as orthologous (Chen et al., 2018). To retrieve the sequences of flanking genes for each locus, the genes closest to an eRTBVL-D locus in the Nipponbare genome and the orthologous genes in the AA-genome species were collected from Gramene (**Supplementary Table 2**). The second flanking gene was used for analysis when the closest ortholog was found in less than five AA-genome species.

### Phylogenetic analysis

**Figure 1:**
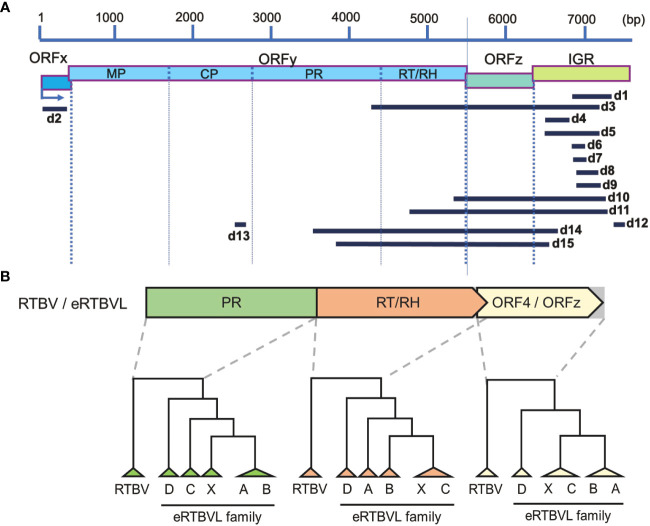
Fifteen segments of the eRTBVL-D family and its evolutionary relationships with the other eRTBVL families and RTBV. **(A)** Fifteen segments of eRTBVL-D (d1-d15) and their alignment with eRTBVL functional regions. Based on eRTBVL sequences, three ORFs (ORFx, y, and z) and an intergenic region (IGR) are predicted within about 7500 bp of eRTBVL sequences. ORFy encodes a polycistronic mRNA encoding movements protein (MP), coat protein (CP), aspartic protease (PR), and reverse transcriptase and RNase H (RT/RH). Most of the eRTBVL-D segment were located between PR and IGR. **(B)** Phylogenetic relationships among RTBV and eRTBVL families (A, B, C, D and X). Phylogenetic trees were built using consensus sequences in three regions: PR, RT/RH, and ORF4/ORFz. ORF4 in RTBV corresponds to ORFz in RTBV and each eRTBVL family.

The phylogenetic relationships of five eRTBVL families, A, B, C, D, and X, were analyzed by constructing phylogenetic trees for three predictable regions in the eRTBVL sequence, a protease (PR) region, a reverse transcriptase/RNase H (RT/RH) region, and an ORFz region (**Figure 1A**). A set of the orthologous sequences within the AA-genome species, such as an eRTBVL-D locus, its flanking sequences, and the closest genes, were used for phylogenetic analysis. The five RTBVL-D sequences from d2, d8, d9, d12, and d13 were omitted from the phylogenetic analysis data sets due to insufficient length or a lack of species carrying the segment (**Supplementary Table 1**). Each of three eRTBVL-D-linked sequences, d4 to d5 (d4-5), d6 to d7 (d6-7), and d10 to d11 (d10-11), which were contained on intervals shorter than 1000 bp, was analyzed as a single locus. In these three loci, the adjacent eRTBVL-D sequences resided on different DNA strands (**Supplementary Table 1**). The sequence alignments of seven data sets (d1, d3, d4-5, d6-7, d10-11, d14, and d15) were generated in MUSCLE (Edgar, 2004), and all gaps were removed. The alignments of the flanking gene sequences were generated with E-INS-i algorithm of MAFFT v.7.490 (Katoh et al., 2019), which is suitable for alignments containing large gaps. Maximum likelihood (ML) phylogenetic analysis was performed, and the best-fitting substitution models for each locus were determined by model selection analysis. ML trees were established based on the support of 1000 bootstrap replicates. All analyses related to phylogenetic analysis were completed using MEGA version 7.0 (Kumar et al., 2016). Evolutionary analyses were conducted in MEGA X (Kumar et al., 2018; Stecher et al., 2020). The numbers of base substitutions per site and variances (analytical method option) were calculated using the Jukes-Cantor model (Jukes and Cantor, 1969; Kimura and Ohta, 1972). All positions containing gaps and missing data were eliminated (complete deletion option).

## Results

### Phylogenetic relationships of eRTBVL families

Fifteen eRTBVL segments found in the *Oryza sativa* ssp. *japonica* (cv. Nipponbare) genome were identified as members of the eRTBVL-D family. These sequences were distinguished from those of previously identified eRTBVL-A, -B, -C, and -X families (Chen et al., 2018). The 15 segments did not contain the entire sequence of the eRTBVL genomes, but corresponded partly or fully to the functional regions in eRTBVL as follows: d2 to ORFx; d13 to coat protein (CP); d3, d14, and d15 to PR; d3, d10, d11, d14, and d15 to reverse transcriptase, RNase H (RT/RH), and ORF4/ORFz; and d1, d3, d4, d5, d6, d7, d8, d9, d10, d11, d12, d14, and d15 to the intergenic region (IGR) (**Figure 1A**). To examine genetic distances among the eRTBVL families, we constructed phylogenetic trees for the PR, RT/RH, and ORF4/ORFz regions using the consensus sequences from the five families and the RTBV representative sequence (**Figure 1B**). Each phylogenetic tree in these three functional regions showed that eRTBVL-D was present in the *Oryza* genomes prior to the integration of the other four eRTBVL families. As Chen et al. (2014; Chen et al., 2018) reported, the phylogenetic tree structures of the eRTBVL-A, -B, -C, and -X families were topologically closely related. The similar structures of the three phylogenetic trees in the PR, RT/RH, and ORFz regions supported the idea that eRTBVL-D sequences existed in the ancestral genome prior to the insertions of the other eRTBVL family members. At four of the loci containing the 15 eRTBVL-D segments (on chromosomes 1, 2, 4, and 7), two or three segments were located within about 1000 bp of the *japonica* genome (cv. Nipponbare) (**Supplementary Figure 1**, **Supplementary Table 1**). We regarded each of these four clusters as an eRTBVL-D locus. Ten positions in the Nipponbare genome were designated as eRTBVL-D loci (**Supplementary Figure 1**).

### Distributions of eRTBVL-D sequences in the AA-genome species

**Figure 2:**
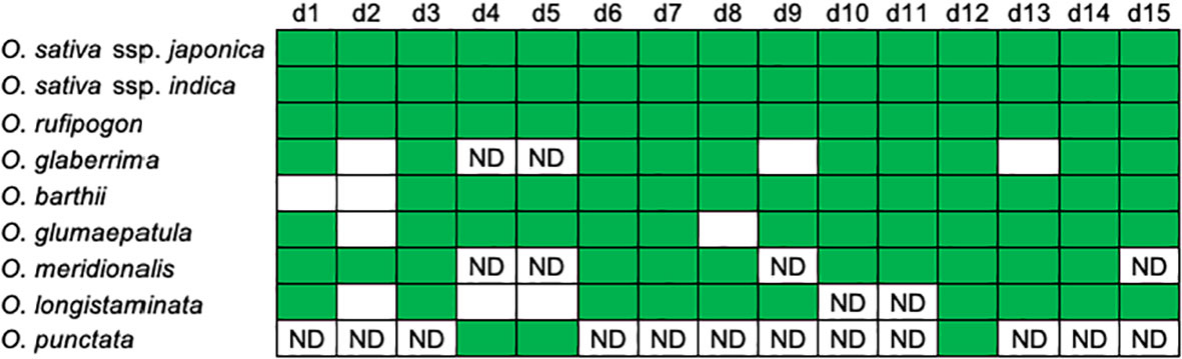
Distributions of the eRTBVL-D segments in the AA-genome species. Patterns of presence (green) or absence (white) of the 15 orthologous eRTBVL-D segments among the AA-genome and O. punctate. This figure was modified from **Figure 1B** of Chen et al. (2018) base on updated data. ND, no definitive significant similarity confirmed in the database.

**Figure 2**; **Supplementary Table 1** summarize characteristics of the orthologous segments of the 15 eRTBVL-D sequences in the nine AA- and BB-genome species and subspecies studied (*O. sativa* ssp. *japonica* and *indica*, *O. rufipogon*, *O. glaberrima*, *O. barthii*, *O. glumaepatula*, *O. meridionalis*, *O. longistaminata*, and *O. punctata*). The d2 sequence was missing in *O. glaberrima*, *O. barthii*, *O. glumaepatula*, and *O. longistaminata*. In *O. longistaminata*, d4 and d5 were lost, and it was unclear whether d10 and d11 were present (**Figure 2**). *O. meridionalis* also contained four ambiguous sequences possibly corresponding to d4, d5, d9, and d15. *O. punctata*, which is the *Oryza* BB-genome species most closely related to the AA-genome species, contained 3 of the 15 eRTBVL-D segments (**Figure 2**; **Supplementary Table 1**). No corresponding sequences were present in a more distant species, *O. brachyantha* (*Oryza* FF-genome) (Chen et al., 2018). Taken together, the patterns of eRTBVL-D distribution in the *Oryza* species strongly support the idea that eRTBVL-D integrations occurred immediately before the AA-genome speciation.

### Phylogenetic relationships of orthologous eRTBVL-D segments

The putative eRTBVL-D sequence was considered to comprise genes or open reading frames (ORFs) similar to those of the other eRTBVL families, which were found in the PR, RT/RH, ORFz, and IGR regions (**Figure 1A**). To examine the phylogenetic relationships of the orthologous eRTBVL-D segments in the AA-genome species, we constructed phylogenetic trees of the eRTBVL-D segments in each of the four representative regions containing such segments. The phylogenetic trees from the PR (d14 and d15), RT/RH (d3, d11, d14, and d15), ORFz (d3, d10, d11, d14, and d15), and IGR (d1, d3, d5, d6, d7, d8, d9, d10, d11) regions displayed mostly clusters consisting of a single orthologous segment (**Supplementary Figure 2**, regions a-f), whereas in the phylogenetic trees of the IGR region, d1/d3 (**Supplementary Figure 2D**) and d10/d11 (**Supplementary Figures 2D–F**) clusters contained different segments. In the d10/11 cluster, the d11 segment from *O. meridionalis* was located in the d10 cluster in the phylogenetic trees for regions d to f in the IGR. The d1/d3 cluster was a mixture of both segments, suggesting that the structural alterations during the AA-genome speciation occurred in the vicinity of the d1-3 locus (**Supplementary Figure 2D**). The five eRTBVL-D segments were excluded from the phylogenetic analyses because they were short and missing the orthologous segments from four or more species.

### Phylogenetic profiles of eRTBVL-D loci among AA-genome species

**Figure 3:**
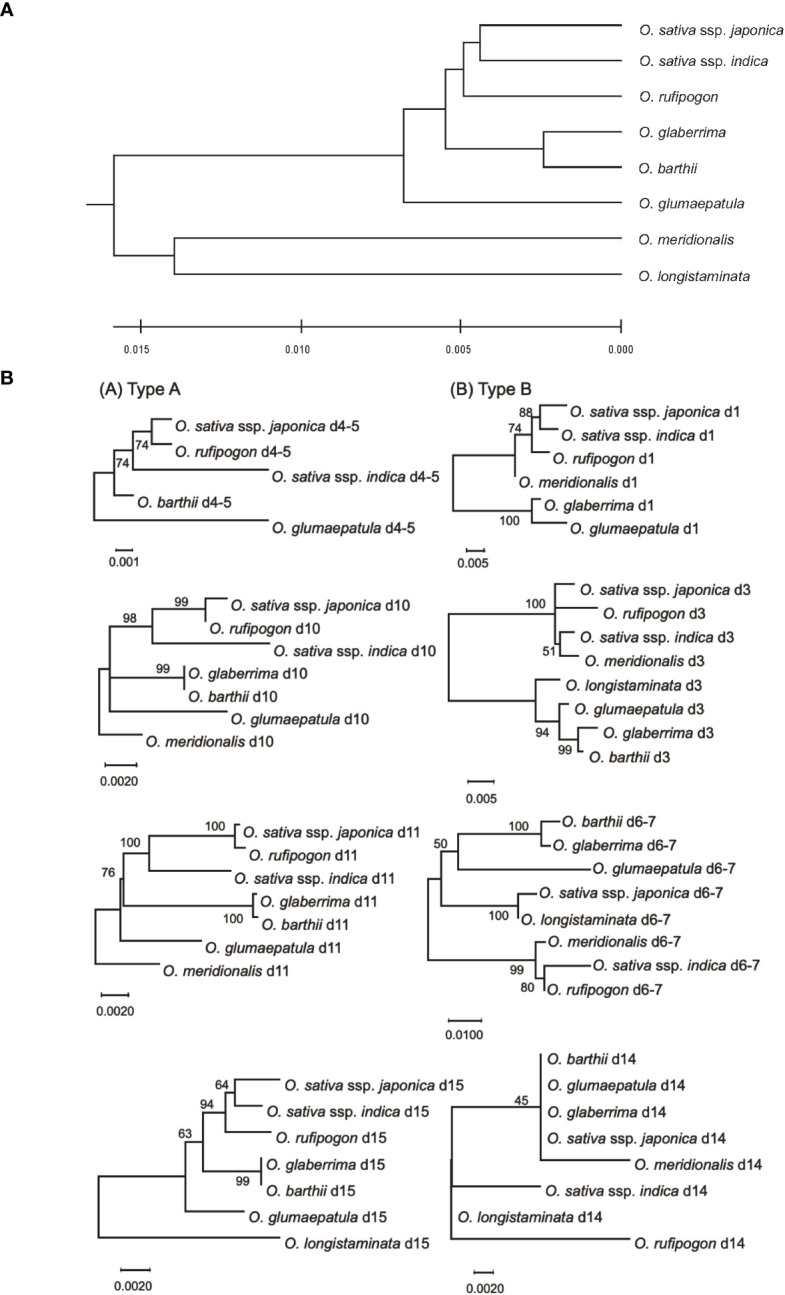
Phylogenetic trees in the AA-genome species based on comprehensive genome data and sequences of eight eRTBVL-D segments. **(A)** The phylogenetic tree contains the six species and two subspecies from the AA-genome species using the genomic sequence data from 53 nuclear genes and 16 intergenic regions (Zhu et al., 2014). The phylogenetic relationships obtained from the tree are designated as the standard specification pattern (SSP). **(B)** The phylogenetic trees of the AA-genome species were constructed using the eight different eRTBVL-D segments. The trees are designated as Type A(d4-5,d10,d11, and d15) and Type B (d1, d3, d6-7, and d14), with the trees in Type A showing topologies similar to the SSP and those in Type B differing from the SSP. Evolutionary history was inferred using the UPGMA method (Sneath and Sokal 1973). The optimal tree with the sum of branch length =0.0589 is shown. The tree is drawn to scale, with branch lengths in the same units as those of the evolutionary distances used to infer the phylogenetic tree. Evolutionary analyses were conducted in MEGA X (Kumar et al., 2018).

Zhu et al. (2014) estimated the genetic distances among the AA-genome species based on the sequences of 53 nuclear genes and 16 intergenic regions. Here, we used these genetic distances and phylogenetic relationships in the eight AA-genome species as the standard speciation pattern (SSP). We compared phylogenetic profiles of eRTBVL-D segments with the SSP of the AA-genome species (Zhu et al., 2014). Two eRTBVL-D loci, d4-5 and d6-7, contained less than 1000 bp of sequence between two eRTBVL-D segments, and each locus contained eRTBVL-D segments on different strands (**Supplementary Table 1**). Each phylogenetic analyses of these two eRTBVL-D loci was performed with the two eRTBVL-D segments containing these interspersed sequences between the segments. We constructed phylogenetic trees of eight eRTBVL-D segments, d1, d3, d4-5, d6-7, d10, d11, d14, and d15, and determined that these trees were classified into two types. Trees constructed from Type A segments (d4-5, d10, d11, and d15) were topologically consistent with the SSP for the eight AA-genome species (**Figures 3A, B**, left). In contrast, analysis of Type B segments (d1, d3, d6-7, and d14) gave rise to a variety of phylogenetic trees for AA-genome species that differed from the SSP (**Figures 3A, B**, right) and that also varied among the four eRTBVL-D segments. The phylogenetic trees derived from d1 and d3 showed that Asian species were in the same clade as the *Oceania* species *O. meridionalis* (**Figures 3B, d1, d3**), whereas the tree from d6-7 identified a species of African origin, *O. longistaminata*, as a sister branch of *O. sativa* ssp. *japonica* with a high bootstrap value (**Figures 3B, d6-7**).

The d14 tree consisted of two clades, each of which contained distantly related species: one for *O. barthii*, *O. glumaepatula*, *O. glaberrima*, *O. sativa* ssp. *japonica*, and *O. meridionalis*, and the other for *O. sativa* ssp. *indica*, *O. longistaminata*, and *O. rufipogon* (**Figures 3B, d14**). Unlike the d6-7 trees, those for d14 indicated low bootstrap values for the relationships among the orthologous sequences due to small numbers of nucleotide polymorphisms (**Figures 3B, d14**). The length of the sequences that could be analyzed was approximately 350 bp for d6-7, which has a sufficient bootstrap value, whereas the d14 sequence was 106 bp but had a lower bootstrap value due to the presence of fewer nucleotide substitutions (**Supplementary Table 1**). The phylogenetic trees from the four Type B eRTBVL-D segments formed specific diverging branches that were apparently inconsistent with the SSP (**Figure 3**). The structure of these four trees caused us to question whether these varied forms were dependent on each specific eRTBVL-D sequence or on the individual chromosomal regions surrounding those sequences.

### Diversity of genetic distances in the eRTBVL-D segments

**Figure 4:**
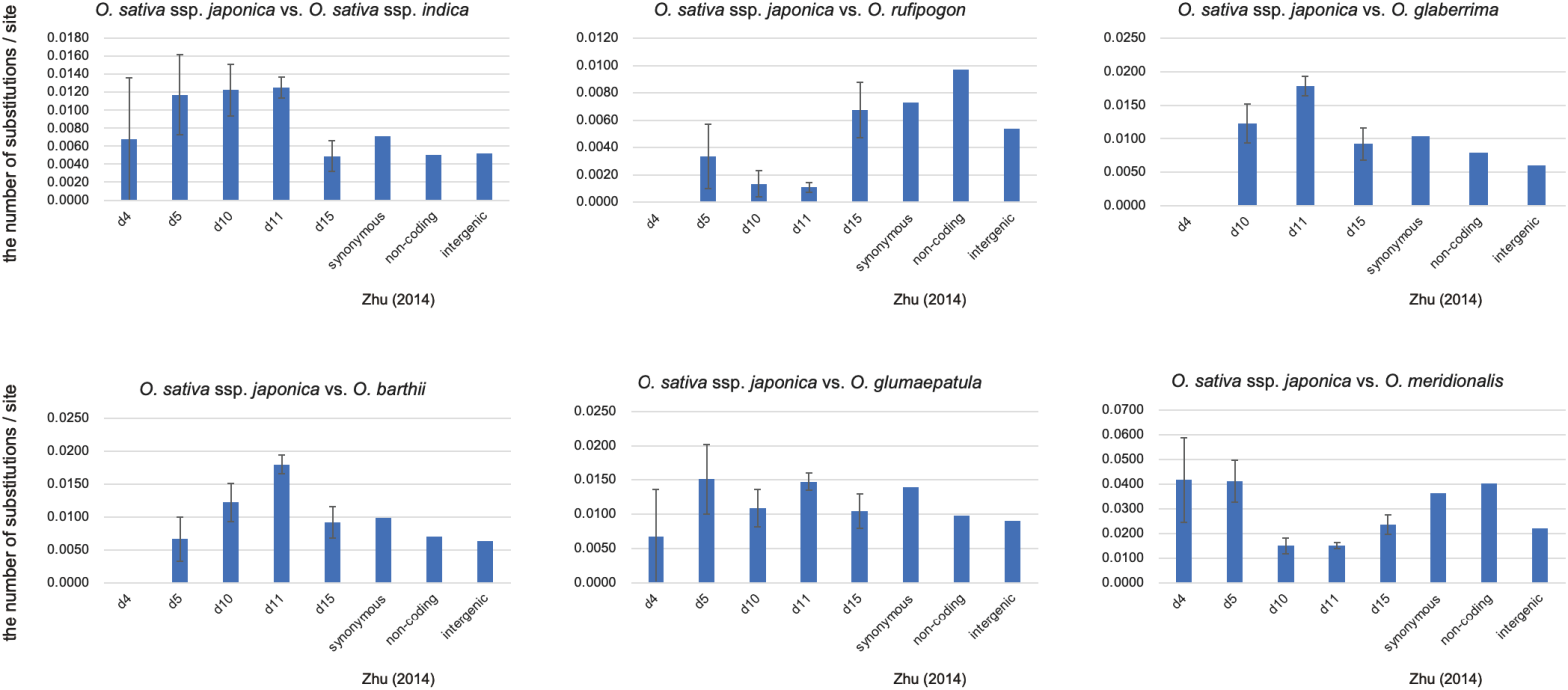
Pairwise evolutionary distances (Jukes-Cantor distances) between O. sativa ssp. Japonica and other Oryza species. The number of base substitutions per site between sequences are shown. Bars represent standard error estimates. Number of sites used for the calculation were 148 bp (d4), 601 bp (d5) 9168 bp (d11), and 1635 bp (d15). Distances of synonymous, non-coding and intergenic regions were taken from Zhu et al. (2014).

Because it is likely that all the eRTBVL-D segments were inserted into the ancestral genome immediately before AA-genome speciation, we observed variations in the genetic distances of different eRTBVL-D segments located on diverse chromosomal regions. We used the phylogenetic tree for the SSP for relative comparison of genetic distances of eRTBVL-D segments between AA-genome species. Type A eRTBVL-D segments (d4-5, d10, d11, and d15) were considered appropriate for evaluating genetic distances (**Figure 3B**, left) because, unlike Type B segments, they did not have aberrant phylogenetic relationships. In each of the four Type A segments, we determined the genetic distances between *O. sativa* ssp. *japonica* and each of the six other species (*O. sativa* ssp. *indica*, *O. rufipogon*, *O. glaberrima*, *O. barthii*, *O. glumaepatula*, and *O. meridionalis*) (**Figure 4**).

**Figure 4** shows the genetic distances of the segments d4, d5, d10, d11, and d15 of Type A eRTBVL-D for each AA-genome species relative to *japonica*. The genetic distances in the AA-genome species analyzed by Zhu et al. (2014) were considered as the standard genetic distances of the SSP. The genetic distances among the five segments detected in *japonica* vs. *glaberrima* and *japonica* vs. *barthii* were different relative to Zhu’s distances. All the genetic distances of the five segments in *japonica* vs. *rufipogon* were smaller than Zhu’s distances. In the remaining three species comparisons vs. *japonica*, the genetic distances were more or less similar to Zhu’s distances. When comparing the genetic distances between each segment and Zhu’s distance, those for d10 and d11 differed from Zhu’s distance, with the exception of *japonica-glumaepatula*. In contrast, the genetic distance of d15 was not detectably different from Zhu’s distance. These results indicate that the genetic distances vs. *japonica* tend to be characteristic of each species to some extent. We found segments such as d15 in eRTBVL-D that evolved with a similar genetic rate as SSP, whereas others, such as d10 and d11, had genetic distances that differed from Zhu’s distances. This demonstrated the existence of chromosomal sites with specific, distinct evolutionary rates of change.

### Effects of the flanking genes on eRTBVL-D loci

To test whether the adjacent genes exhibited the same evolutionary patterns as the eRTBVL-D sequences, we constructed phylogenetic trees using the genes flanking each eRTBVL-D sequence. Because the eRTBVL sequences of d1, d2, and d3 were clustered on chromosome 1 (**Supplementary Figure 1**), we analyzed all three as a single locus. The flanking gene sequences were obtained from the regions upstream of d1 and downstream of d3 in *japonica*. This was also the case for d10 and d11 on chromosome 7 (**Supplementary Figure 1**), which we considered as a single region for the phylogenetic analysis. Detailed information for the flanking genes is shown in **Supplementary Table 2**.

**Figure 5:**
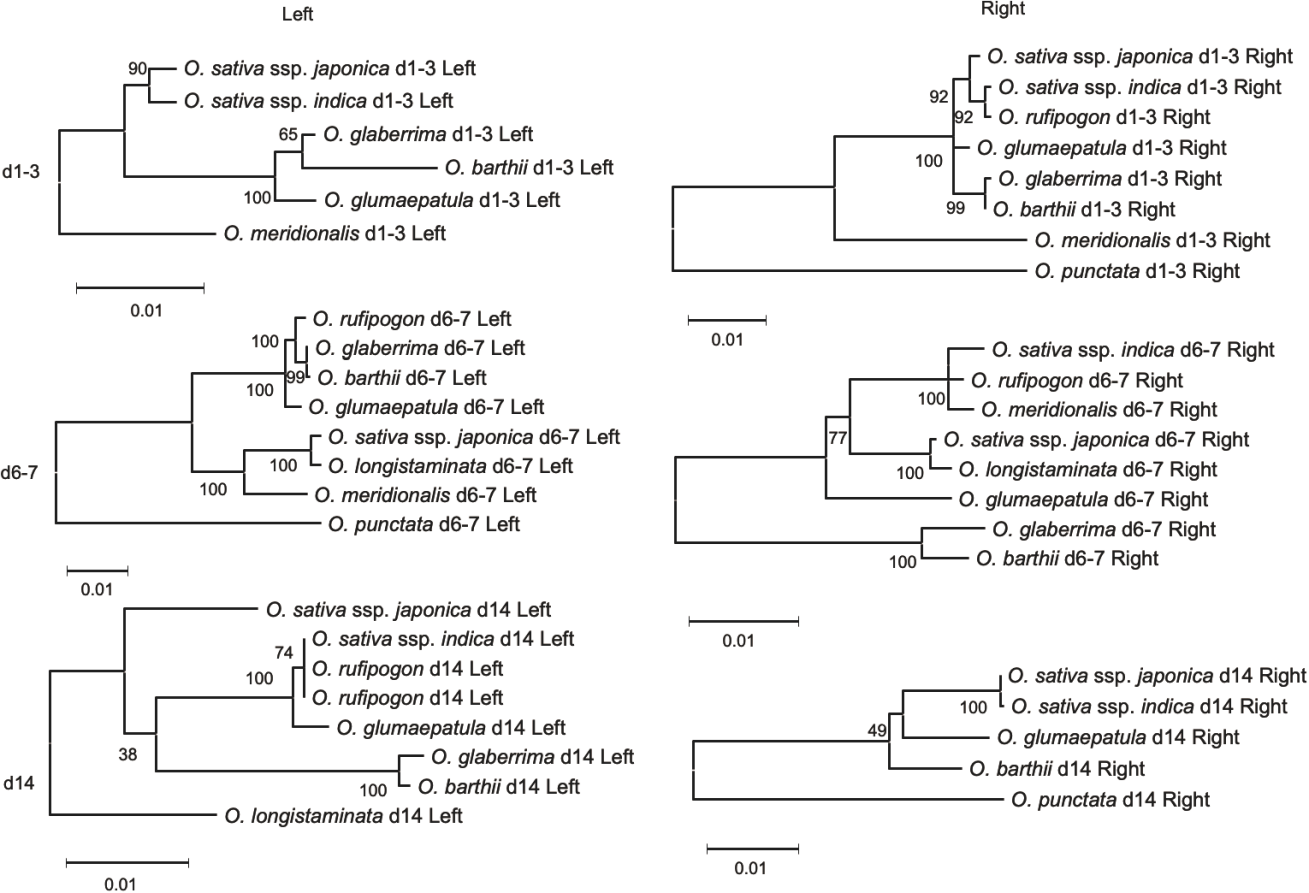
Phylogenetic tress based on flanking genes of type B eRTBVL-D segments. Numbers represent bootstrap supports. Number of sites used for the inference were 948 bp (d1-3 Left), 3713 bp (d6-7 Left), 1074 bp (d14 Left), 1491 bp (d1-3 Right), 2106 bp (d6-7 Right) and 2610 bp (d14 Right).

The phylogenetic trees for the genes flanking d4-5, d10-11, and d15, classified in Type A, showed a similar phylogenetic relationship with the SSP for the AA-genome species (**Supplementary Figure 3**). The flanking genes surrounding the d1-3 locus in Type B exhibited phylogenies different from those of the corresponding eRTBVL-D segments. For example, the d1-3 segment may have exclusively differentiated during AA-genome speciation relative to the neighboring regions (**Figures 5, d1-3**). The eRTBVL-D segments of d1-3 contained sequences homologous to each other, and these might represent paralogous relationships. The flanking genes of the Type B loci d6-7 and d14 exhibited similar phylogenetic trees with respect to their eRTBVL-D segments, but these also were not consistent with that of the SSP (**Figures 5, d6-7, d14**). This suggests that each of the chromosomal regions containing the d6-7 and d14 segments is likely to have evolved independently from the SSP. As observed for the eRTBVL-D segments themselves (**Figure 3**), the bootstrap values were relatively high for the flanking genes of d6-7 but were relatively low for the genes near d14 (**Figure 5**).

### Rearrangements of the region containing the eRTBVL-D locus

**Figure 6:**
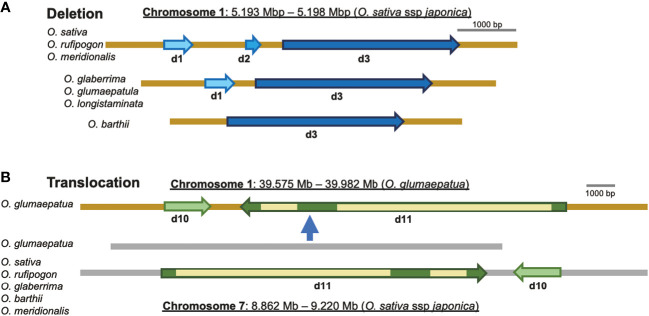
Rearrangements of the local chromosomal regions, including eRTBVL-D segments. **(A)** Deletions: the regions containing d1, d2, and d3 ranging from 5.193 to 5. 198 Mb on chromosome 1 (O. sativa ssp. Japonica) showed different deletions in different AA-genome species. O. sativa, O. rufipogon, and O. meridionalis had three eRTBVL-D segments, d1 d2, and d3, in the region. O. glaberrima, O. glumaepatula, and O. longistaminata possessed two segments, d1, d2. O. barthii had only the d3 segments. **(B)** Translocation: two segments, d10 and d11, located between 8.862 and 9.220 Mb in chromosome 7 (O. sativa ssp. Japonica) were found in most of the AA-genome species, whereas in O. glumaepatula, the intact region containing d10 and d11 (but in the reverse orientation) was translocated from chromosome 7 to the region from 39.575 to 39.982 Mb on chromosome 1.

Chromosomal rearrangements such as insertions/deletions (indels) and translocations have been found to be associated with some eRTBVL-D segments, and these are presumed to have occurred during speciation of the AA-genome species. We compared the chromosomal structures around eRTBVL-D loci of the *japonica* (Nipponbare) genome with those of the other AA-genome species. Chromosome 1 in *japonica* retained three eRTBVL sequences, d1, d2, and d3, and these have three different alignments among the AA-genome species. Although the d2 sequence between d1 and d3 was present in *O. sativa*, *O. rufipogon*, and *O. meridionalis*, it was absent from the genomes of *O. glaberrima*, *O. glumaepatula*, and *O. longistaminata* (**Figures 2**, **6**). In *O. barthii*, only d3 is present, and the d1 and d2 segments have been lost (**Figures 2**, **6**). Notably, *O. glaberrima* shared phylogenetic structure not with its direct ancestor, *O. barthii*, but instead with *O. longistaminata*. Both *O. barthii* and *O. longistaminata* originated from Africa, even though they did not originate from the same lineage according to the SSP (**Figure 3A**). These rearrangements of the d1-3 segments could have caused an alteration of the phylogenetic lineage from the SSP of the AA-genome species.

Most of the AA-genome species possessed a similar structure in the region surrounding the d10-11 segment present in chromosome 7. An interchromosomal rearrangement, i.e., a translocation of the region containing d10-11 and a gene adjacent to chromosome 1, was observed for *O. glumaepatula* (**Figure 6**). This translocation contained the 350- to 400-kbp region including the two eRTBVL-D segments (**Figure 6**). The presence of the eRTBVL-D segments substantially facilitated our detection of the genomic rearrangements. These local chromosomal rearrangements occurred in the eRTBVL-D sequences and might have occurred in other chromosomal regions in the AA-genome species. They can be discovered unless the sequences containing them share the same origin. In regard to phylogenetic relationship, the d10-11 segments were grouped into Type A along with the SSP, suggesting that the translocation did not affect the phylogenetic relationships of d10-11 segments among the AA-genome species (**Figures 3B, d10, d11**, **6**).

## Discussion

### Endogenization of eRTBVL-D sequences prior to the AA-genome speciation

eRTBVL-D is a group of viruses that were active in the early stages of differentiation of the AA-genome species of the genus *Oryza*, as evidenced by the common distribution of EVEs in the genomes of all AA-genome species (Chen et al., 2014; Chen et al., 2018). The *O. punctata* genome, which is the *Oryza* BB-genome species most closely related to the AA-genome group, contains three possible eRTBVL-D segments (d4, d5, and d12) of the 15, but we found no clear homologous sequences for the other 12 segments (**Figure 2**). As it is likely that the integration of eRTBVL-D occurred in an active period of virus infection (Chen et al., 2018), each eRTBVL-D segment may have been inserted into the ancestral AA-genome species just prior to its differentiation into distinct species and thus was inherited by all current AA-genome species.

Because the other eRTBVL families are present only in the genomes of *O. sativa* and *O. rufipogon*, eRTBVL-D is probably the ancestral virus of the entire eRTBVL family (Chen et al., 2018). eRTBVL-A, -B, -C, -X, and -D are more closely related to each other than to the current active virus, RTBV (**Figure 1B**) (Chen et al., 2014). RTBV infects rice via the green leafhoppers. If eRTBVL-D was a similar virus, it was possible that it was transmitted via vector-borne insects. Previous analyses of the eRTBVL families have shown that insertions of each family into the genome occurred almost simultaneously during their active periods (Chen et al., 2014; Chen and Kishima, 2016). Based on their structural features, Chen et al. (2014) identified the evolutionary timing and order of insertion of each family of eRTBVL into *O. sativa* ssp. *japonica*, *indica*, and *O. rufipogon*. Their results indicated that each family was inserted during a limited period of the speciation process. Geering et al. (2014) newly classified Florendovirus from the Caulimoviridae, to which the eRTBVL family belongs, and associated the occurrence of Florendoviruses with the lineages of the host plant genus. Relationships between speciation and virus endogenization have been reported in *Musa* (Ndowora et al., 1999), tomato (Strasburg et al., 2012), yam (Seal et al., 2014), *Dahlia* (Almeyda et al., 2014), and *Citrus* (Yu et al., 2019) species, with virus generation and endogenization being correlated with speciation in each case.

Multiple genome databases are available for *japonica* and *indica*, while for the other AA-genome species, few sequences other than those used here are publicly available. The present study aims to investigate whether the local chromosomal regions had specific evolutionary movements. Therefore, it is conceivable that using multiple genomic data from *japonica* and *indica* would provide similar answers for the respective eRTBVL-D copies in speciation.

### Variation of genetic distances of Type A segments among the AA-genome species

As shown in **Figure 4**, the genetic distances between the Type A segments of *japonica* and other species varied among AA-genome species. These results also implied that the evolutionary rates varied depending on the different positions of the segments in the respective chromosome. As reported by Chen et al. (2018), eRTBVL segments are assumed to have undergone neutral evolution after insertion; therefore, the genetic distances observed can be considered to directly reflect the mutation rates. **Figure 4** and **Supplementary Table 3** show the genetic distances between *japonica* and the other AA-genome species for the five Type A segments, d4, d5, d10, d11, and d15. For comparison with neutrally evolving sequences of corresponding species, standard genetic distances for synonymous, noncoding, and intergenic regions analyzed by Zhu et al. (2014) are also shown in **Figure 4**. The different genetic distances among Type A loci in the same combination with *japonica* suggested the existence of variation in mutation rates for the different genomic regions. Relative to Zhu’s distances (2014), the genetic distances for each segment between *japonica* and other species also showed inconsistency, implying a variation in mutation rates for different lineages. In this way, eRTBVL segments can be utilized as evolutionary indicators of variations in local chromosomal regions.

### Phylogenetic incongruence regarding Type B segments

Phylogenetic relationships of the Type B segments have different topologies from the SSP (**Figure 3B**, right). Phylogenetic incongruence can be caused not only by biological factors such as violations of orthology due to lineage sorting, hidden paralogy, and horizontal gene transfer but also by analytical factors such as violation of assumed evolutionary models (Som, 2015). Genomic factors in the violations were often related to major biological changes associated with evolutionary movements, such as introgression (Cronn et al., 2003; Eaton and Ree, 2013), genetic drift (Tomiuk et al., 1998), meiotic drive (Presgraves, 2010), adaptation (Strasburg et al., 2012; Bamba et al., 2019), and horizontal transmission (MacLeod et al., 2005; Bailly et al., 2007). The phylogenetic data collected by Zhu et al. (2014) has revealed more than 20 topologies in cases where they used 53 nuclear genes and 16 intergenic regions among the AA-genome species. Thus, in the process of analyzing phylogenetic trees, stochastic incongruence of a topology might be caused by rapid radiation of genes or intergenic regions. These factors could explain what we observed in the phylogenetic relationships of Type B segments (d1, d3, d6-7, and d14) (**Figure 3B**, right), as each of the Type B segments exhibited some of the characteristics associated with phylogenetic incongruence.

#### Structural rearrangements of d1 and d3 resulted in phylogenetic incongruence

The phylogenetic trees of eRTBVL-D segments d1 and d3 differed from those surrounding genes and the SSP (**Figures 3B, d1, d3**; **Figures 5, d1-3** Left and Right). The *O. sativa*/*rufipogon*/*meridionalis* groups have three segments, d1, d2, and d3, whereas the *O. glaberrima*/*longistaminata/glumaepatula* group lacks d2, and *O. barthii* lacks d1 and d2 (**Figure 6A**). The phylogenetic trees of the AA-genome species for both d1 and d3 loci clearly separated the group with the three segments from the other two groups (**Figures 3B, d1, d3**). *O. meridionalis* was included in the *japonica*/*indica* clade in the trees for both d1 and d3, whereas the SSP shows that *O. meridionalis* was extremely distant from *O. sativa*/*rufipogon* among the AA-genome species (**Figure 3A**). Genomic rearrangement itself does not influence phylogenetic relationships, but gene duplication following gene loss and gene conversion caused by rearrangements could hinder orthologous relationship of corresponding loci, resulting in apparent alteration of the phylogenetic tree topology from the SSP (Som, 2015). The proximity of d1, d2, and d3 and their structural variation (deletion of d2 in some lineages) could be attributed to rearrangements between or among these homologous sequences. In addition, we have provided evidence for the hidden paralogy of d1 and d3 segments where the two clades comprise these mixed segments (**Supplementary Figures 2D–F**). Although it is unclear how the structural rearrangements in d1, d2, and d3 occurred during AA-genome speciation, the phylogenetic incongruence found here indicates a complex evolutionary history for these loci.

#### Introgression detected by eRTBVL sequences

The d6-7 and d14 Type B segments exhibited unusual phylogenetic differentiation and evolved in parallel with the flanking regions (**Figure 5**). Analysis of these chromosomal segments provided evidence that the local chromosomal regions have synchronously differentiated in some of the phylogenetic relationships within the genera. We propose that introgression was involved in the aberrant phylogenetic relationships in d6-7. *O. sativa* and *O. longistaminata* might have exchanged some genomic regions after the two species had differentiated. The evidence for the introgression between the two species was obtained from the phylogenetic tree of d6-7 where *japonica* and *O. longistaminata* were closely related (**Figures 3B, d6-7**). The eRTBVL families other than eRTBVL-D are likely to have been active as viruses in Asia because they were incorporated into genomes of the Asian species during their differentiation (Chen et al., 2014). The vectors of the eRTBVL families would have lived in Asia, like the rice green leafhopper (*Nephotettix cincticeps*), the insect vector for the existing virus, RTBV (Dale, 1994; Hohn, 2013). Our previous experimental results demonstrated that the eRTBVL sequences were detected more often in the Asian species *O. sativa* and *O. rufipogon*; fairly often in the Oceanian species *O. meridionalis* and one African species, *O. longistaminata*; and least often in two other African species, *O. barthii* and *O. glaberrima*, and a Latin American species, *O. glumaepatula* (Kunii et al., 2004) (**Supplementary Figure 5**). African and Latin American species might not have come into contact with the insect vectors carrying the viruses containing the eRTBVL segments, resulting in the fewer numbers of eRTBVL sequences in the genomes of *O. barthii*, *O. glaberrima*, and *O. glumaepatula*. Even though no virus vector was present, the African species *O. longistaminata* had a certain number of the eRTBVL segments in the genome (**Supplementary Figure 5**). This implies that the genomic regions with the eRTBVL segments in Asian species were introgressed into *O. longistaminata* after these species were differentiated. The phylogenetic trees of the d6-7 locus provide evidence for the introgression of the genomic region from *O. longistaminata* to Asian species (**Figures 3B, d6-7**).

#### Incongruence with low genetic diversity

The phylogenetic incongruence in d14 might be attributed to the low bootstrap values, which were caused by the presence of a small number of nucleotide substitutions. In any large amount of phylogenetic data, there must be many potential genomic regions that have a different topology from that of the SSP due to an insufficient amount of genetic mutation (Strasburg et al., 2012). Removing *O. meridionalis* d14, which is short, increased the number of sites used for phylogenetic inference from 106 to 553 bp. This raised the bootstrap values, but the tree topology was still different from SSP (**Supplementary Figure 4B**). We found insertion/deletions in the d14 sequence that clearly distinguished *O. barthii*, *O. glumaepatula*, *O. glaberrima*, and *O. sativa* ssp. *japonica* from *O. sativa* ssp. *indica*, *O. longistaminata*, and *O. rufipogon*, which is consistent with the tree topology (**Supplementary Figure 3A**). Although we do not know the mechanism(s) for the incongruence, d14 resides in a chromosomal region containing a dense cluster of F-box genes and histone deacetylase-related genes (**Supplementary Figure 6**), which might have brought complicated evolutionary history like that found in d1-3 locus. Here, we demonstrated the genetic differentiation of the local chromosomal regions and quantified the genetic changes from the early stage of speciation in the AA-genome species using a set of eRTBVL-D sequences with clear origins.

## Conclusions

Among the 15 segments of eRTBVL-D inserted at roughly the same time shortly before AA-genome speciation, we analyzed the eRTBVL-D segments distributed in six regions and categorized them into Type A segments, which differentiated according to the AA-genome speciation, and Type B segments, which did not follow the standard speciation pattern of the AA-genome species (Type A SSP; **Figure 3**). The phylogenetic patterns of Type A segments revealed that local chromosome regions could have given rise to varying degrees of genetic distance between the AA-genome species (**Figure 4**). Rearrangements such as translocations likely did not influence topological variation among the AA-genome species (**Figures 3B, d10-11**, **6B**). Three possible reasons for the unique phylogenetic differentiations in Type B eRTBVL-D loci are as follows: 1) the hidden paralogue given by rearrangements among the homologous elements; 2) introgression between two species exchanging local chromosomal segments may have caused the phylogenetic trees to differ from the SSP, and the results of this study suggest the recent incorporation of *japonica* genome fragments within the genome of *O. longistaminata*; and 3) an incongruence in topology of the local chromosomal region during speciation may have caused it to diverge from the SSP. This study revealed that rearrangements among paralogous sequences, introgression, and stochastic lineages due to lack of genetic variation are responsible for region-specific phylogenetic relationships. In conclusion, we showed that EVEs such as eRTBVL-D segments incorporated into the genome at the starting point of speciation identify locations on chromosomes showing unusual phylogenetic relationships and topologies during speciation.

## Data availability statement

The datasets presented in this study can be found in online repositories. The names of the repository/repositories and accession number(s) can be found in the article/**Supplementary Material**.

## Author contributions

NS: Conceptualization, Data curation, Formal Analysis, Investigation, Writing – original draft. SC: Conceptualization, Data curation, Investigation, Methodology, Resources, Formal Analysis, Writing – review & editing. KK: Formal Analysis, Investigation, Methodology, Writing – review & editing. ZZ: Data curation, Formal Analysis, Methodology, Resources, Visualization, Writing – review & editing. YKo: Supervision, Validation, Writing – review & editing. JE: Methodology, Validation, Writing – review & editing. MD: Supervision, Validation, Writing – review & editing. IC: Project administration, Supervision, Validation, Writing – review & editing. KK: Data curation, Methodology, Validation, Visualization, Writing – original draft, Writing – review & editing. YKi: Conceptualization, Funding acquisition, Project administration, Supervision, Validation, Writing – original draft, Writing – review & editing.

## References

[B1] Almeyda C. V. Eid S. G. Saar D. Samuitiene M. Pappu H. R. (2014). Comparative analysis of endogenous plant pararetroviruses in cultivated and wild Dahlia spp. Virus Genes 48 (1), 140–152. doi: 10.1007/s11262-013-0997-9 24353027

[B2] Bailly X. Olivieri I. Brunel B. Cleyet-Marel J. C. Bena G. (2007). Horizontal gene transfer and homologous recombination drive the evolution of the nitrogen-fixing symbionts of Medicago species. J. Bacteriology 189 (14), 5223–5236. doi: 10.1128/Jb.00105-07 PMC1951869 17496100

[B3] Bamba M. Kawaguchi Y. W. Tsuchimatsu T. (2019). Plant adaptation and speciation studied by population genomic approaches. Dev. Growth Differ 61 (1), 12–24. doi: 10.1111/dgd.12578 30474212

[B4] Brozynska M. Copetti D. Furtado A. Wing R. A. Crayn D. Fox G.. (2017). Sequencing of Australian wild rice genomes reveals ancestral relationships with domesticated rice. Plant Biotechnol. J. 15 (6), 765–774. doi: 10.1111/pbi.12674 27889940 PMC5425390

[B5] Chabannes M. Caruana M. L. I. (2013). Endogenous pararetroviruses - a reservoir of virus infection in plants. Curr. Opin. Virol. 3 (6), 615–620. doi: 10.1016/j.coviro.2013.08.012 24035682

[B6] Chen S. Kishima Y. (2016). Endogenous pararetroviruses in rice genomes as a fossil record useful for the emerging field of palaeovirology. Mol. Plant Pathol. 17 (9), 1317–1320. doi: 10.1111/mpp.12490 27870389 PMC6638417

[B7] Chen S. Liu R. Koyanagi K. O. Kishima Y. (2014). Rice genomes recorded ancient pararetrovirus activities: Virus genealogy and multiple origins of endogenization during rice speciation. Virology 471-473, 141–152. doi: 10.1016/j.virol.2014.09.014 25461539

[B8] Chen S. Saito N. Encabo J. R. Yamada K. Choi I. R. Kishima Y. (2018). Ancient endogenous pararetroviruses in oryza genomes provide insights into the heterogeneity of viral gene macroevolution. Genome Biol. Evol. 10 (10), 2686–2696. doi: 10.1093/gbe/evy207 30239708 PMC6179347

[B9] Chu H. Jo Y. Cho W. K. (2014). Evolution of endogenous non-retroviral genes integrated into plant genomes. Curr. Plant Biol. 1, 55–59. doi: 10.1016/j.cpb.2014.07.002

[B10] Cronn R. Small R. L. Haselkorn T. Wendel J. F. (2003). Cryptic repeated genomic recombination during speciation in Gossypium gossypioides. Evolution 57 (11), 2475–2489. doi: 10.1111/j.0014-3820.2003.tb01493.x 14686525

[B11] Dale D. (1994). “Insect pests of the rice plant - their biology and ecology,” in Biology and Management of Rice Pests. Ed. Heinrichs E. A. (Los Baños, Laguna, Phillipines: International Rice Research Institute and Wiley Eastern Ltd), 363–486.

[B12] Eaton D. A. R. Ree R. H. (2013). Inferring phylogeny and introgression using RADseq data: an example from flowering plants (Pedicularis: orobanchaceae). Systematic Biol. 62 (5), 689–706. doi: 10.1093/sysbio/syt032 PMC3739883 23652346

[B13] Edgar R. C. (2004). MUSCLE: multiple sequence alignment with high accuracy and high throughput. Nucleic Acids Res. 32 (5), 1792–1797. doi: 10.1093/nar/gkh340 15034147 PMC390337

[B14] Feschotte C. Gilbert C. (2012). Endogenous viruses: insights into viral evolution and impact on host biology. Nat. Rev. Genet. 13 (4), 283–U288. doi: 10.1038/nrg3199 22421730

[B15] Geering A. D. W. Maumus F. Copetti D. Choisne N. Zwickl D. J. Zytnicki M.. (2014). Endogenous florendoviruses are major components of plant genomes and hallmarks of virus evolution. Nat. Commun. 5, 5269. doi: 10.1038/ncomms6269 25381880 PMC4241990

[B16] Harper G. Hull R. Lockhart B. Olszewski N. (2002). Viral sequences integrated into plant genomes. Annu. Rev. Phytopathol. 40, 119–136. doi: 10.1146/annurev.phyto.40.120301.105642 12147756

[B17] Harper G. Osuji J. O. Heslop-Harrison J. S. Hull R. (1999). Integration of banana streak badnavirus into the Musa genome: Molecular and cytogenetic evidence. Virology 255 (2), 207–213. doi: 10.1006/viro.1998.9581 10069945

[B18] Hohn T. (2013). Plant pararetroviruses: interactions of cauliflower mosaic virus with plants and insects. Curr. Opin. Virol. 3 (6), 629–638. doi: 10.1016/j.coviro.2013.08.014 24075119

[B19] Hull R. (1996). Molecular biology of rice tungro viruses. Annu. Rev. Phytopathol. 34, 275–297. doi: 10.1146/annurev.phyto.34.1.275 15012544

[B20] Hull R. Harper G. Lockhart B. (2000). Viral sequences integrated into plant genomes. Trends Plant Sci. 5 (9), 362–365. doi: 10.1016/s1360-1385(00)01723-4 11203277

[B21] Jakowitsch J. Mette M. F. van der Winden J. Matzke M. A. Matzke A. J. M. (1999). Integrated pararetroviral sequences define a unique class of dispersed repetitive DNA in plants. Proc. Natl. Acad. Sci. United States America 96 (23), 13241–13246. doi: 10.1073/pnas.96.23.13241 PMC23932 10557305

[B22] Jukes T. H. Cantor C. R. (1969). “Evolution of protein molecules,” in Mammalian Protein Metabolism. Ed. M.H. N. (New York: Academic Press), 21–132.

[B23] Katoh K. Rozewicki J. Yamada K. D. (2019). MAFFT online service: multiple sequence alignment, interactive sequence choice and visualization. Brief Bioinform. 20 (4), 1160–1166. doi: 10.1093/bib/bbx108 28968734 PMC6781576

[B24] Kim H. San Miguel P. Nelson W. Collura K. Wissotski M. Walling J. G.. (2007). Comparative physical mapping between Oryza sativa (AA genome type) and O. punctata (BB genome type). Genetics 176 (1), 379–390. doi: 10.1534/genetics.106.068783 17339227 PMC1893071

[B25] Kimura M. Ohta T. (1972). On the stochastic model for estimation of mutational distance between homologous proteins. J. Mol. Evol. 2, 4. doi: 10.1007/BF01653945 4668865

[B26] Kumar S. Stecher G. Li M. Knyaz C. Tamura K. (2018). MEGA X: molecular evolutionary genetics analysis across computing platforms. Mol. Biol. Evol. 35 (6), 1547–1549. doi: 10.1093/molbev/msy096 29722887 PMC5967553

[B27] Kumar S. Stecher G. Tamura K. (2016). MEGA7: molecular evolutionary genetics analysis version 7.0 for bigger datasets. Mol. Biol. Evol. 33 (7), 1870–1874. doi: 10.1093/molbev/msw054 27004904 PMC8210823

[B28] Kunii M. Kanda M. Nagano H. Uyeda I. Kishima Y. Sano Y. (2004). Reconstruction of putative DNA virus from endogenous rice tungro bacilliform virus-like sequences in the rice genome: implications for integration and evolution. BMC Genomics 5, 80. doi: 10.1186/1471-2164-5-80 15488154 PMC526188

[B29] Liu R. Koyanagi K. O. Chen S. Kishima Y. (2012). Evolutionary force of AT-rich repeats to trap genomic and episomal DNAs into the rice genome: lessons from endogenous pararetrovirus. Plant J. 72 (5), 817–828. doi: 10.1111/tpj.12002 22900922

[B30] MacLeod D. Charlebois R. L. Doolittle F. Bapteste E. (2005). Deduction of probable events of lateral gene transfer through comparison of phylogenetic trees by recursive consolidation and rearrangement. BMC Evolutionary Biol. 5, 27. doi: 10.1186/1471-2148-5-27 PMC1087482 15819979

[B31] Nagel J. H. Wingfield M. J. Slippers B. (2021). Next-generation sequencing provides important insights into the biology and evolution of the Botryosphaeriaceae. Fungal Biol. Rev. 38, 25–43. doi: 10.1016/j.fbr.2021.09.002

[B32] Ndowora T. Dahal G. LaFleur D. Harper G. Hull R. Olszewski N. E.. (1999). Evidence that badnavirus infection in Musa can originate from integrated pararetroviral sequences. Virology 255 (2), 214–220. doi: 10.1006/viro.1998.9582 10069946

[B33] Peterson-Burch B. D. Wright D. A. Laten H. M. Voytas D. F. (2000). Retroviruses in plants? Trends Genet. 16 (4), 151–152. doi: 10.1016/s0168-9525(00)01981-8 10729827

[B34] Presgraves D. C. (2010). The molecular evolutionary basis of species formation. Nat. Rev. Genet. 11 (3), 175–180. doi: 10.1038/nrg2718 20051985

[B35] Richert-Poggeler K. R. Noreen F. Schwarzacher T. Harper G. Hohn T. (2003). Induction of infectious petunia vein clearing (pararetro) virus from endogenous provirus in petunia. EMBO J. 22 (18), 4836–4845. doi: 10.1093/emboj/cdg443 12970195 PMC212712

[B36] Seal S. Turaki A. Muller E. Kumar P. L. Kenyon L. Filloux D.. (2014). The prevalence of badnaviruses in West African yams (Dioscorea cayenensis-rotundata) and evidence of endogenous pararetrovirus sequences in their genomes. Virus Res. 186, 144–154. doi: 10.1016/j.virusres.2014.01.007 24457074

[B37] Sneath P. H. A. Sokal R. R. (1973). Numerical taxonomy: The principles and practice of numerical classification. (San Francisco, USA: Freeman & Co.), 573p.

[B38] Som A. (2015). Causes, consequences and solutions of phylogenetic incongruence. Briefings Bioinf. 16 (3), 536–548. doi: 10.1093/bib/bbu015 24872401

[B39] Staginnus C. Richert-Poggeler K. R. (2006). Endogenous pararetroviruses: two-faced travelers in the plant genome. Trends Plant Sci. 11 (10), 485–491. doi: 10.1016/j.tplants.2006.08.008 16949329

[B40] Stecher G. Tamura K. Kumar S. (2020). Molecular evolutionary genetics analysis (MEGA) for macOS. Mol. Biol. Evol. 37 (4), 1237–1239. doi: 10.1093/molbev/msz312 31904846 PMC7086165

[B41] Stein J. C. Yu Y. Copetti D. Zwickl D. J. Zhang L. Zhang C.. (2018). Genomes of 13 domesticated and wild rice relatives highlight genetic conservation, turnover and innovation across the genus Oryza. Nat. Genet. 50 (2), 285–296. doi: 10.1038/s41588-018-0040-0 29358651

[B42] Strasburg J. L. Sherman N. A. Wright K. M. Moyle L. C. Willis J. H. Rieseberg L. H. (2012). What can patterns of differentiation across plant genomes tell us about adaptation and speciation? Philos. Trans. R Soc. Lond B Biol. Sci. 367 (1587), 364–373. doi: 10.1098/rstb.2011.0199 22201166 PMC3233712

[B43] Tomiuk J. Guldbrandtsen B. Loeschcke V. (1998). Population differentiation through mutation and drift - a comparison of genetic identity measures. Genetica 102-3, 545–558. doi: 10.1023/A:1017080119277 9720297

[B44] Yu H. Wang X. Lu Z. Xu Y. Deng X. Xu Q. (2019). Endogenous pararetrovirus sequences are widely present in Citrinae genomes. Virus Res. 262, 48–53. doi: 10.1016/j.virusres.2018.05.018 29792903

[B45] Zhang J. Chen L. L. Xing F. Kudrna D. A. Yao W. Copetti D.. (2016). Extensive sequence divergence between the reference genomes of two elite indica rice varieties Zhenshan 97 and Minghui 63. Proc. Natl. Acad. Sci. U.S.A. 113 (35), E5163–E5171. doi: 10.1073/pnas.1611012113 27535938 PMC5024649

[B46] Zhu T. Xu P. Z. Liu J. P. Peng S. Mo X. C. Gao L. Z. (2014). Phylogenetic relationships and genome divergence among the AA- genome species of the genus Oryza as revealed by 53 nuclear genes and 16 intergenic regions. Mol. Phylogenet Evol. 70, 348–361. doi: 10.1016/j.ympev.2013.10.008 24148990

[B47] Zimmer E. A. Wen J. (2012). Using nuclear gene data for plant phylogenetics: Progress and prospects. Mol. Phylogenet. Evol. 65 (2), 774–785. doi: 10.1016/j.ympev.2012.07.015 22842093

[B48] Zimmer E. A. Wen J. (2015). Using nuclear gene data for plant phylogenetics: Progress and prospects II. Next-gen approaches. J. Systematics Evol. 53 (5), 371–379. doi: 10.1111/jse.12174

