# Supplementary material for: Phylogenetic analysis of endogenous viral elements in the rice genome reveals local chromosomal evolution in Oryza AA-genome species
Source: Front Plant Sci. 2023 Oct 27;14:1261705. doi: 10.3389/fpls.2023.1261705 (PMC10641527; doi:10.3389/fpls.2023.1261705)
Supplement: Supplementary file 2 [file DataSheet_1.pdf]

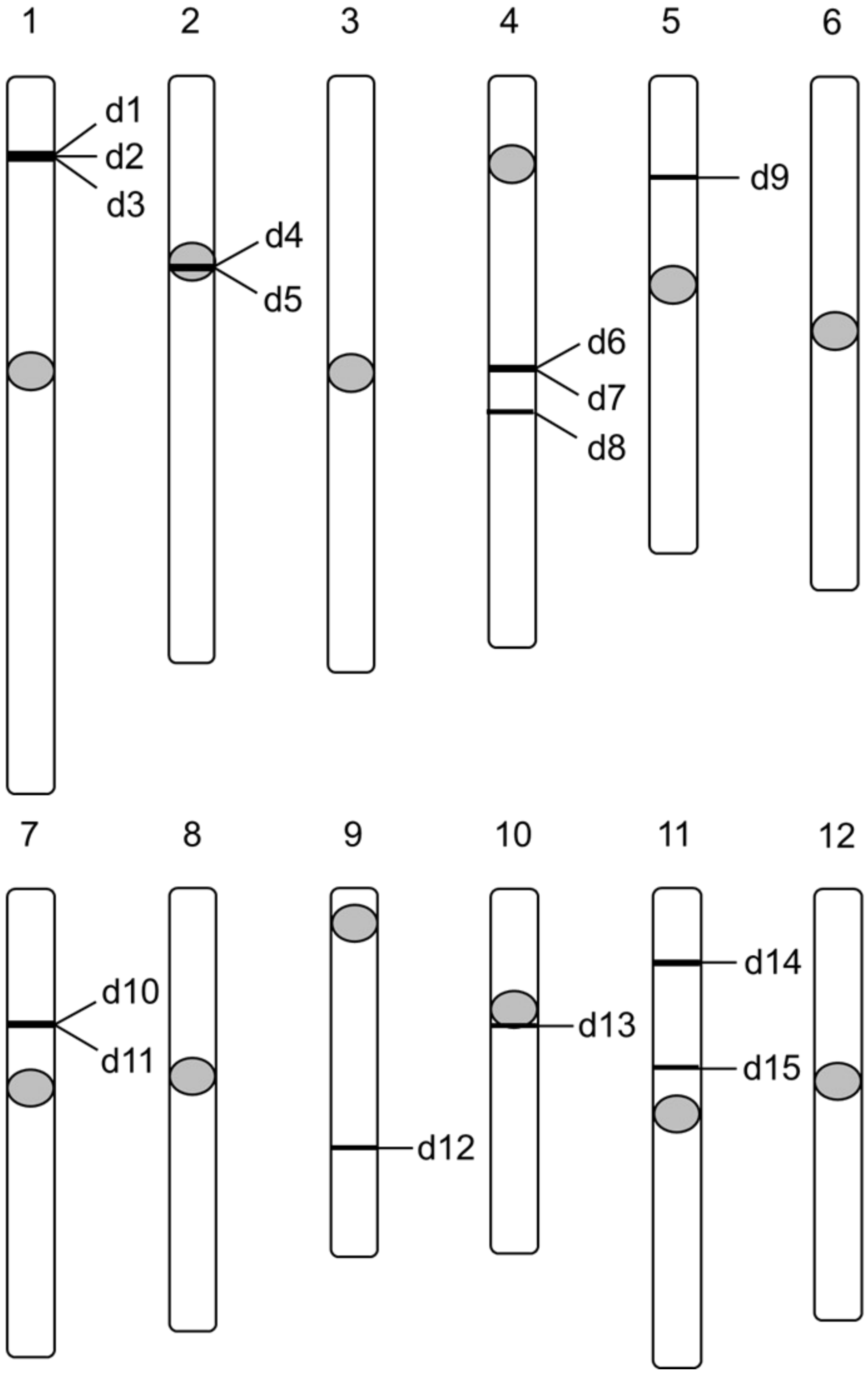

Supplementary Figure 1
Chromosomal positions of the 15 eRTBVL-D segments in the *O. sativa* ssp. *japonica* genome.
The 15 eRTBVL-D segments are distributed among 10 loci on eight different chromosomes (1, 2, 4, 5, 7, 9, 10, and 11), with four of these loci containing more than two segments.

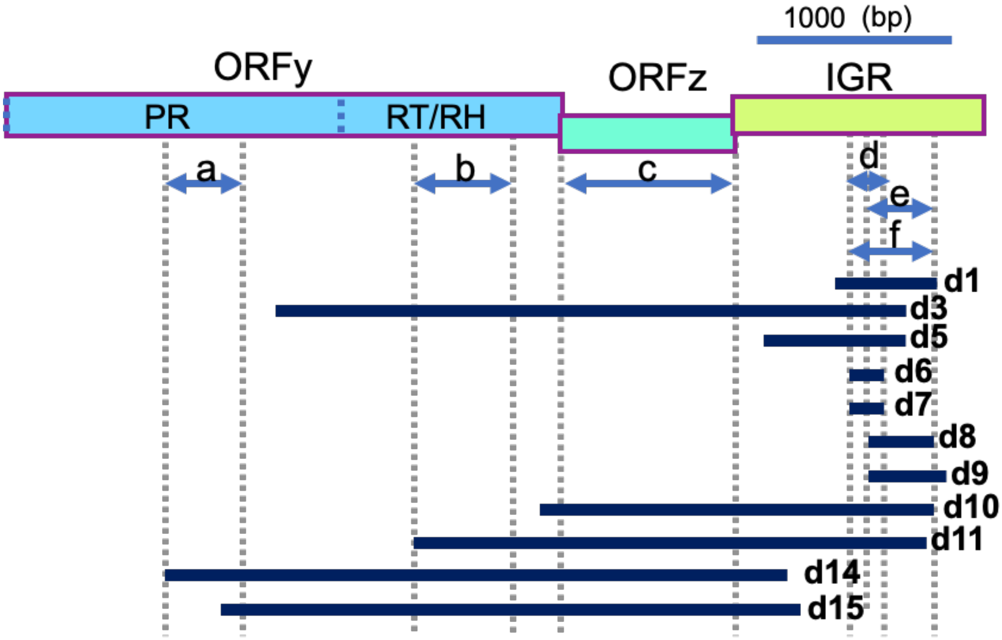

Supplementary Figure 2
Phylogenetic trees of the six regions (a to f) in the representative eRTBVL-D sequence constructed using sequences of all the corresponding segments found in the AA-genome species.
Above, the functional regions of the eRTBVL sequence, with the corresponding regions of the eRTBVL-D segments, d1 to d15, indicated by blue lines. Below, the phylogenetic trees for regions a to f.

Supplementary Figure 3

a

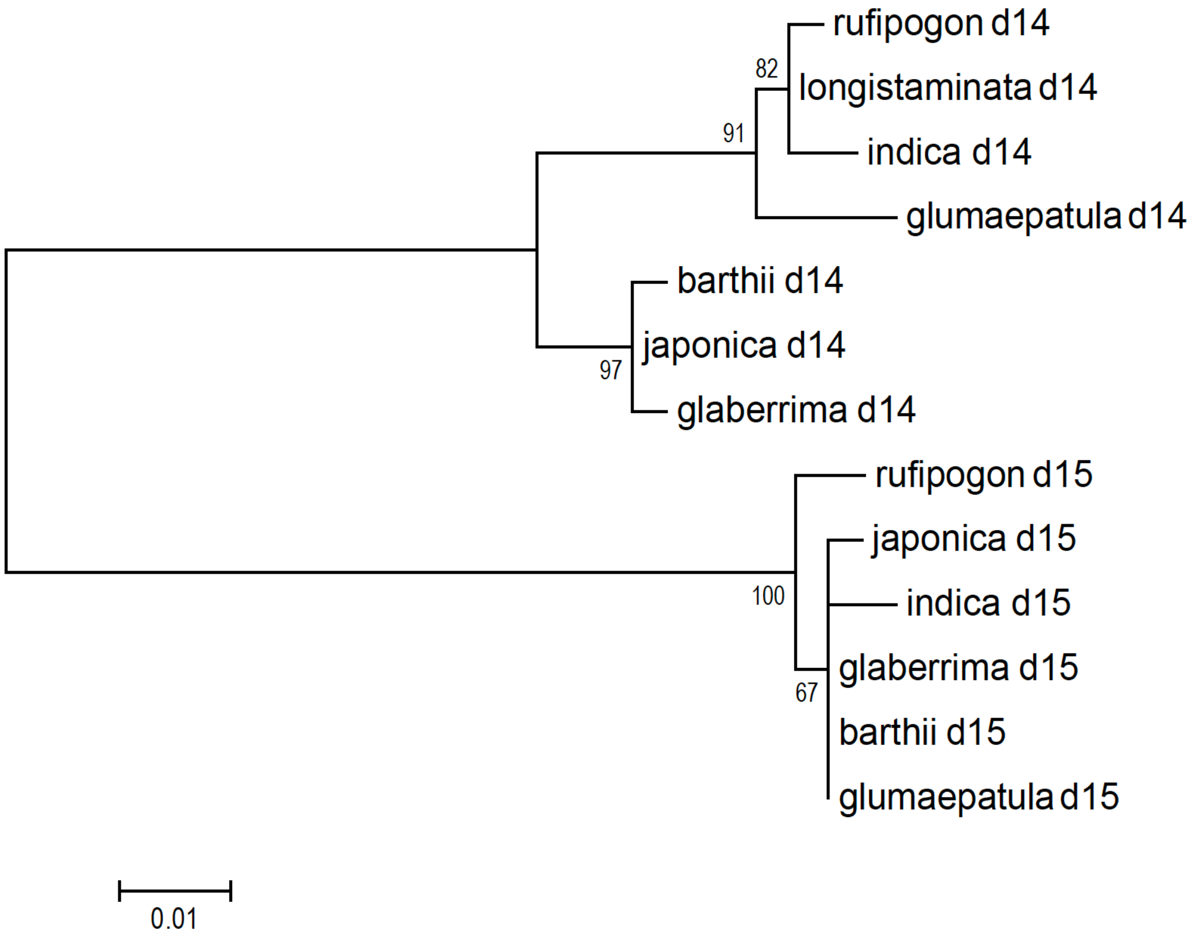

Supplementary Figure 3

b

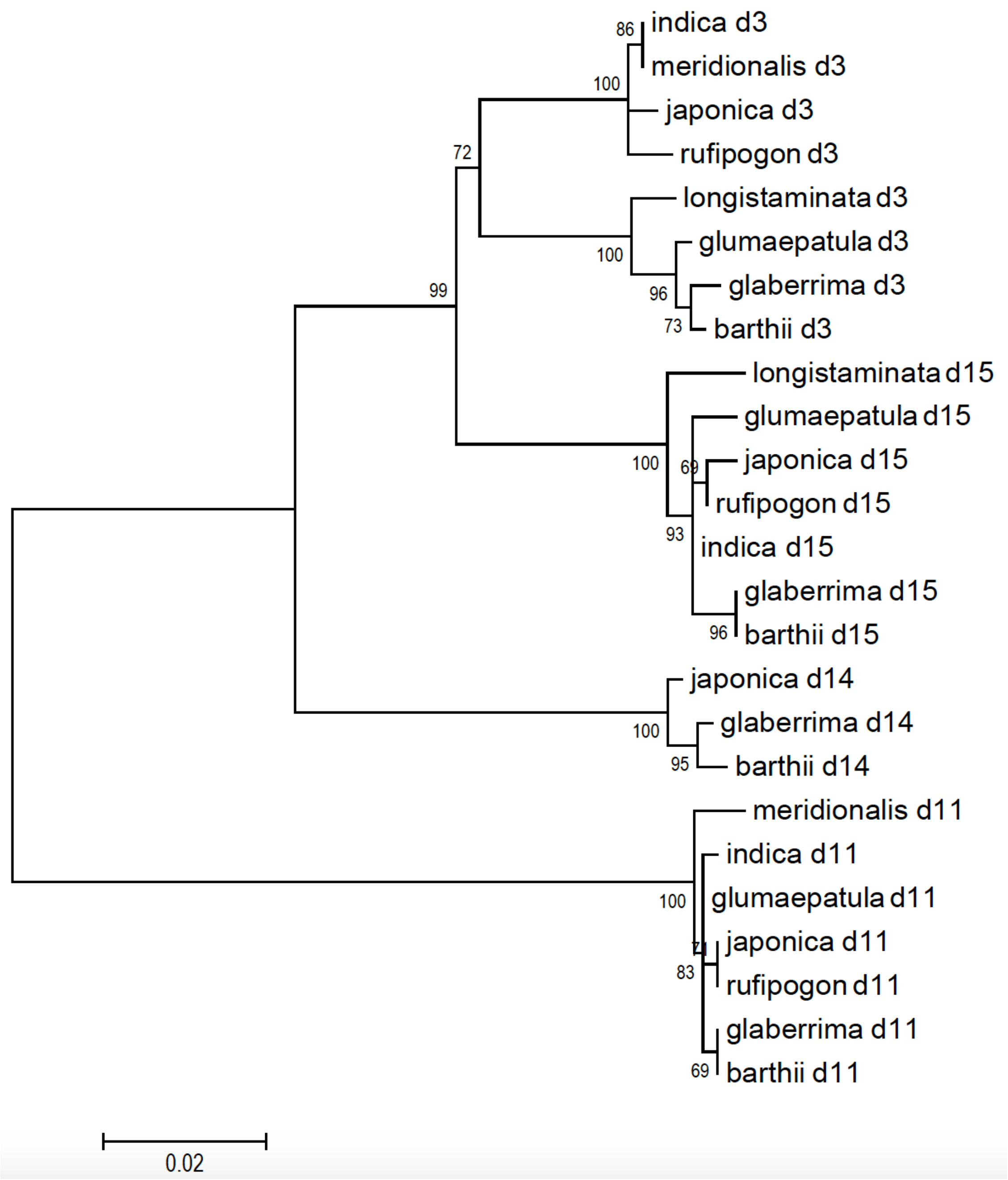

Supplementary Figure 3

c

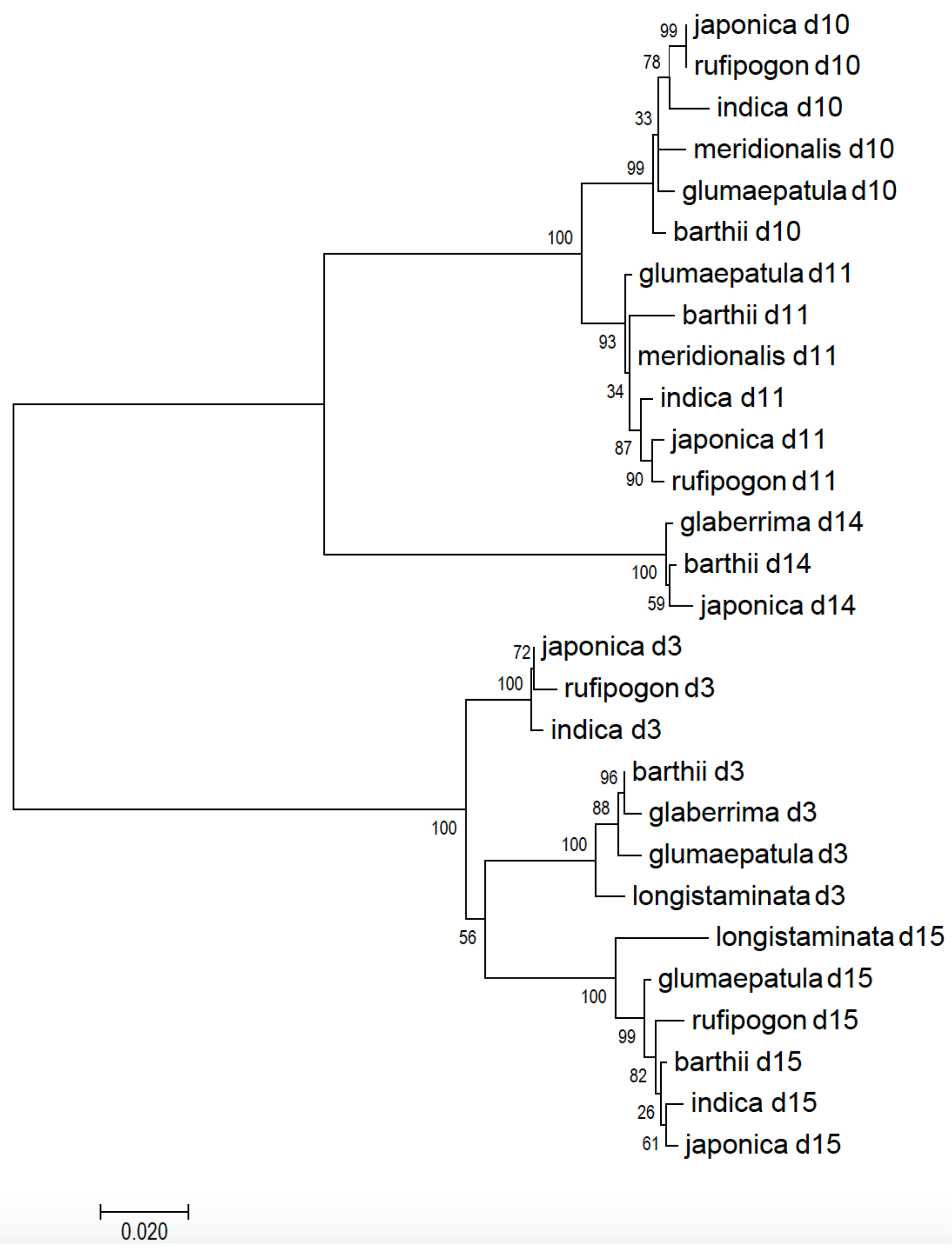

Supplementary Figure 3

d

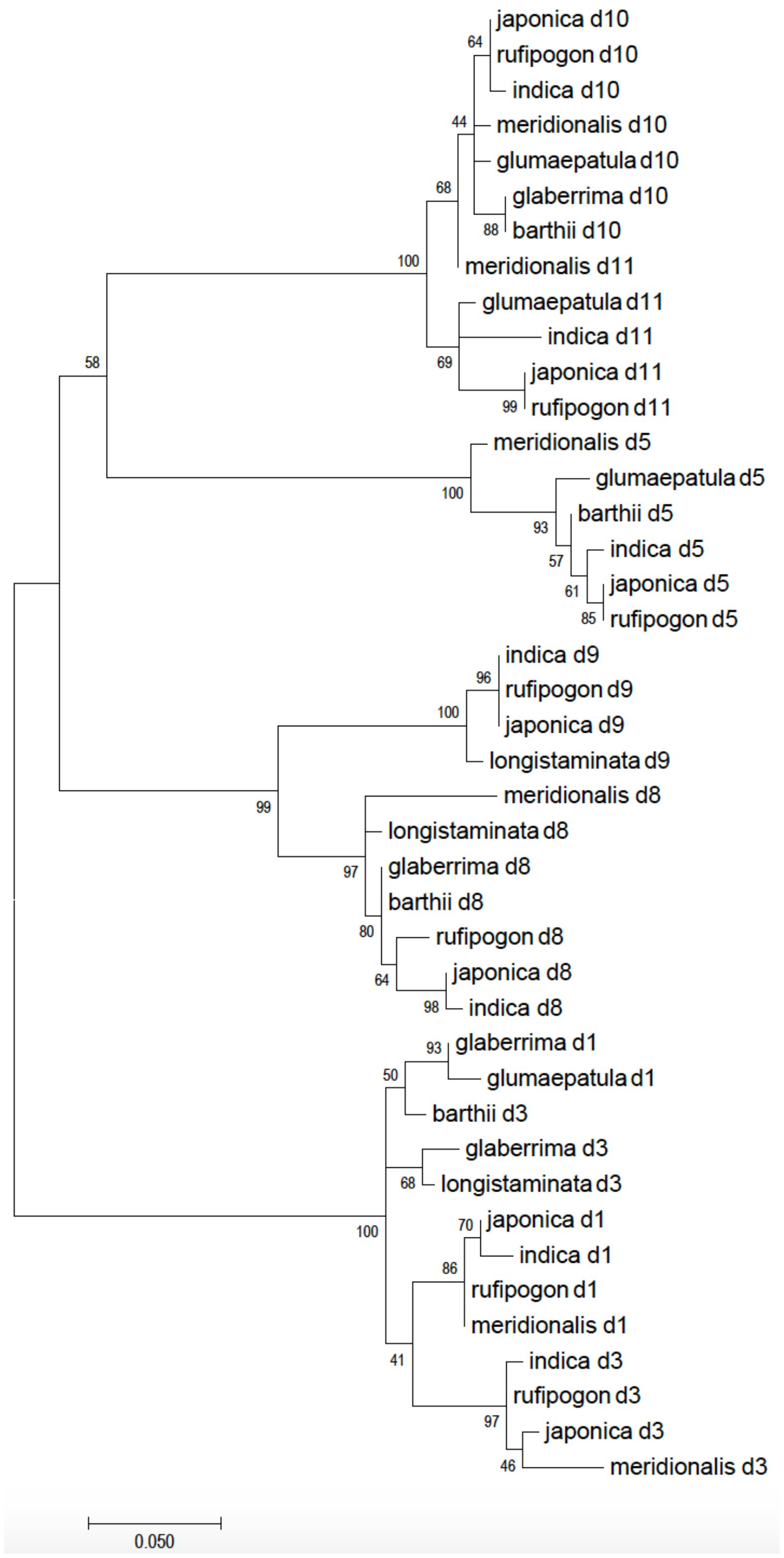

Supplementary Figure 3

e

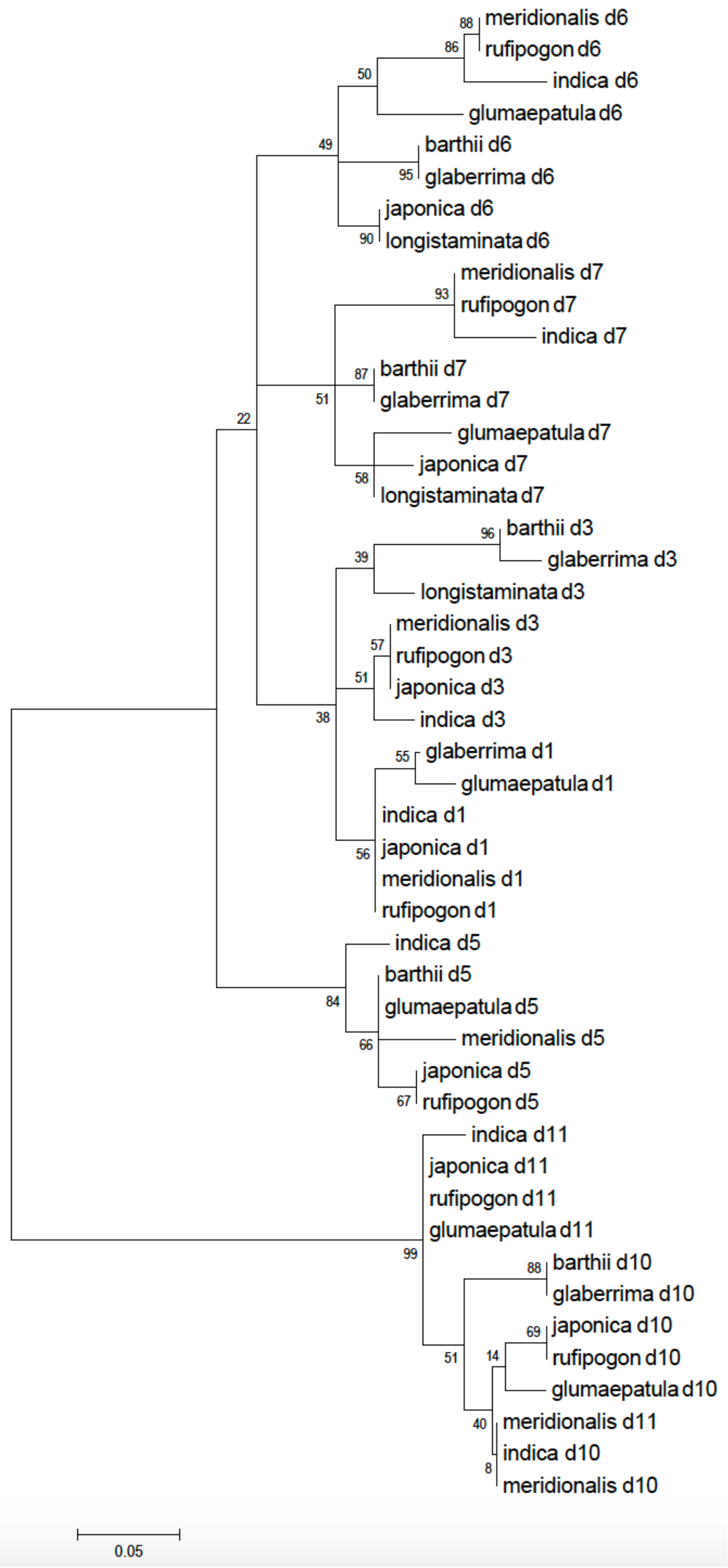

Supplementary Figure 3

f

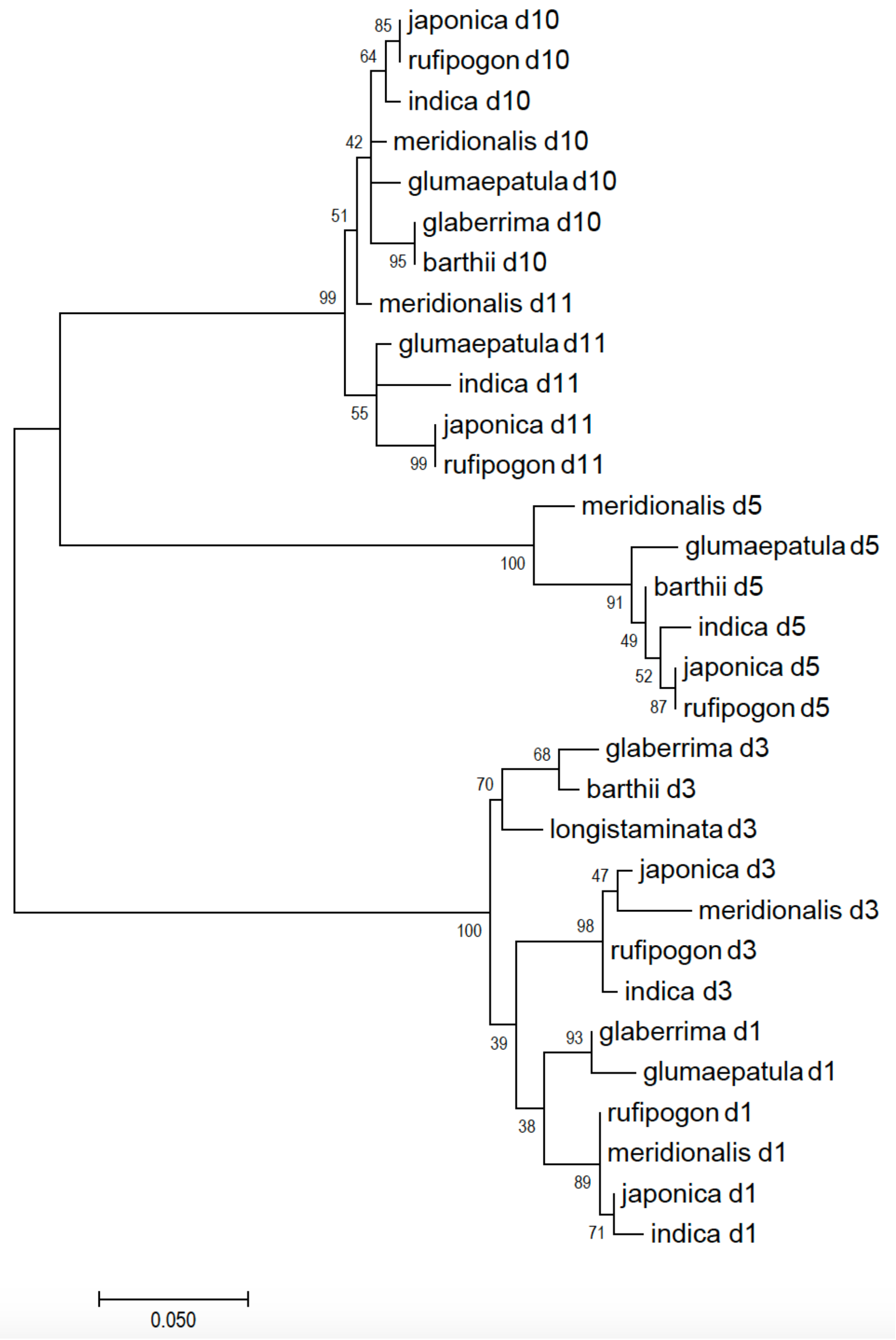

Supplementary Figure 3

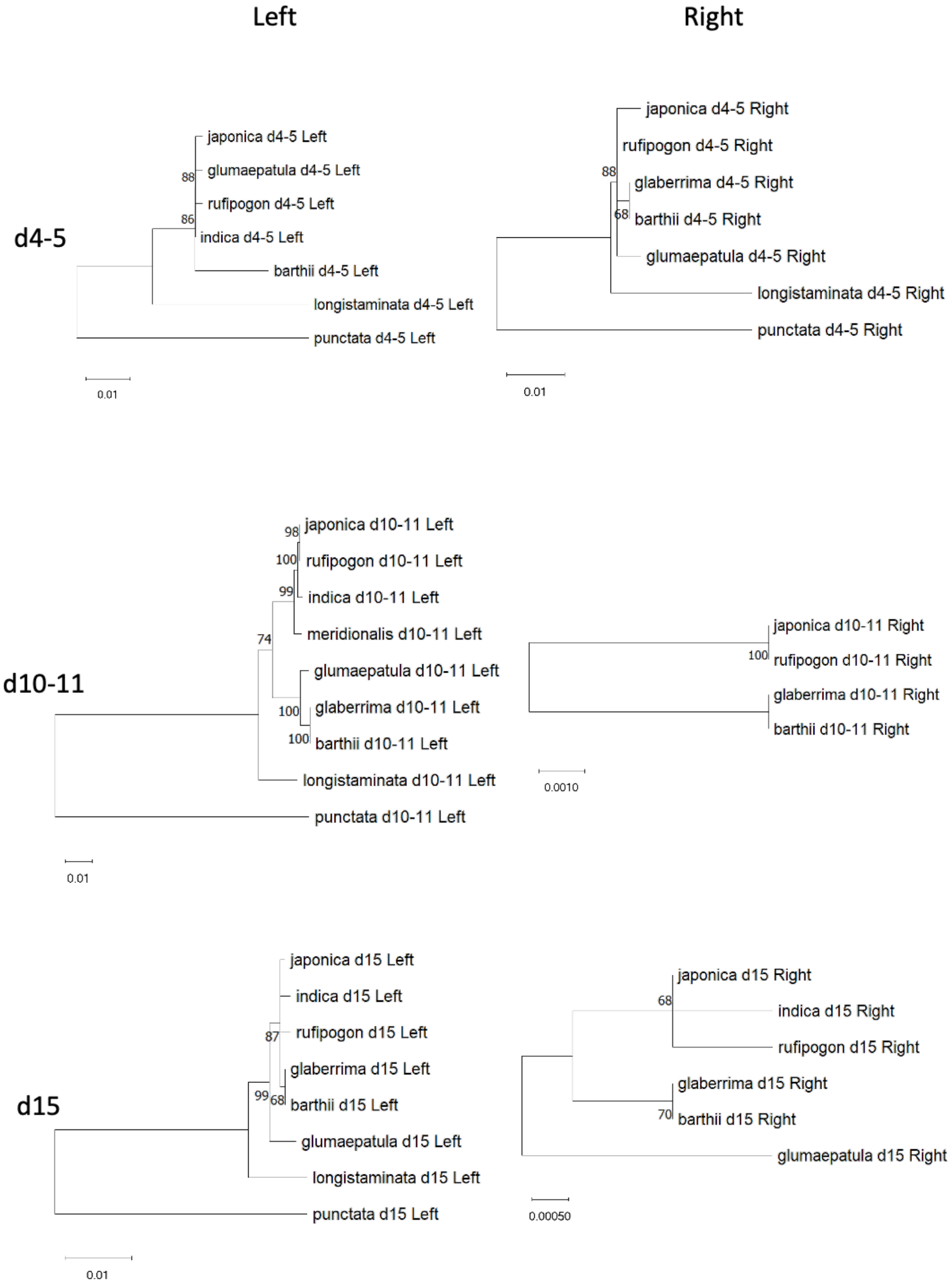

Supplementary Figure 3
Phylogenetic trees based on sequences of genes flanking Type A eRTBVL-D segments.
Numbers represent bootstrap supports. Number of sites used for the inference were 603 bp (Left, d4-5), 4281 bp (Left, d10-11), 1263 bp (Left, d15), 483 bp (Right, d4-5), 2421 bp (Right, d10-11), and 729 bp (Right, d15).

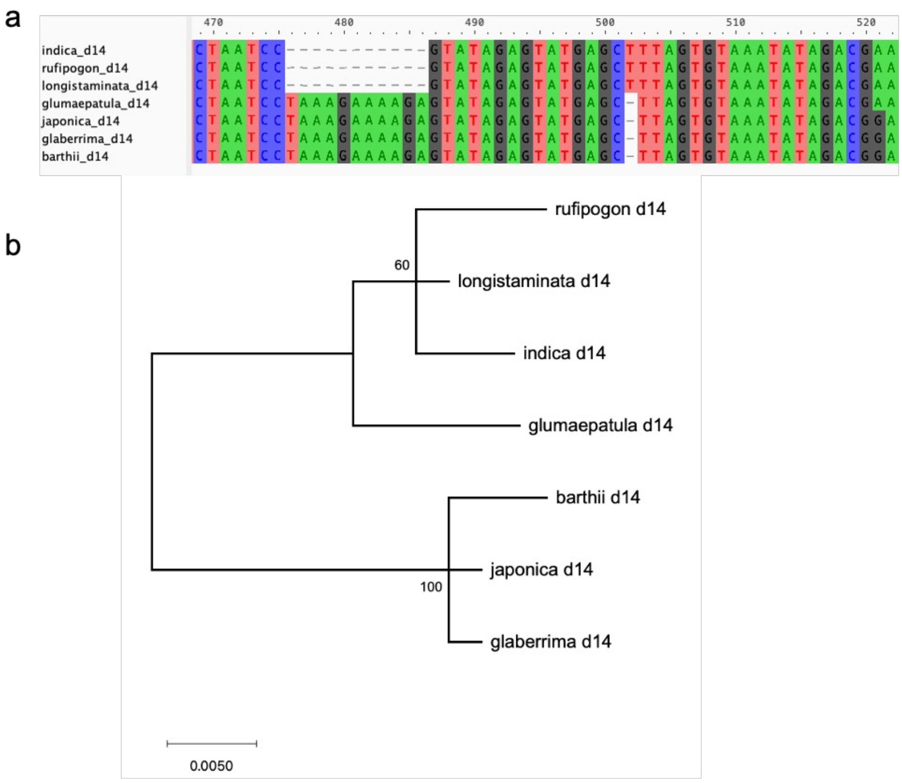

Supplementary Figure 4

An alignment of d14 segment sequences on chromosome 11 and a corresponding phylogenetic tree.

a: A region of chromosome 11 containing cluster-specific indels. b: A phylogenetic tree representing seven subspecies after removing *O. meridionalis*. The number of sites used for the inference was 553 bp. Numbers at tree nodes represent bootstrap supports.

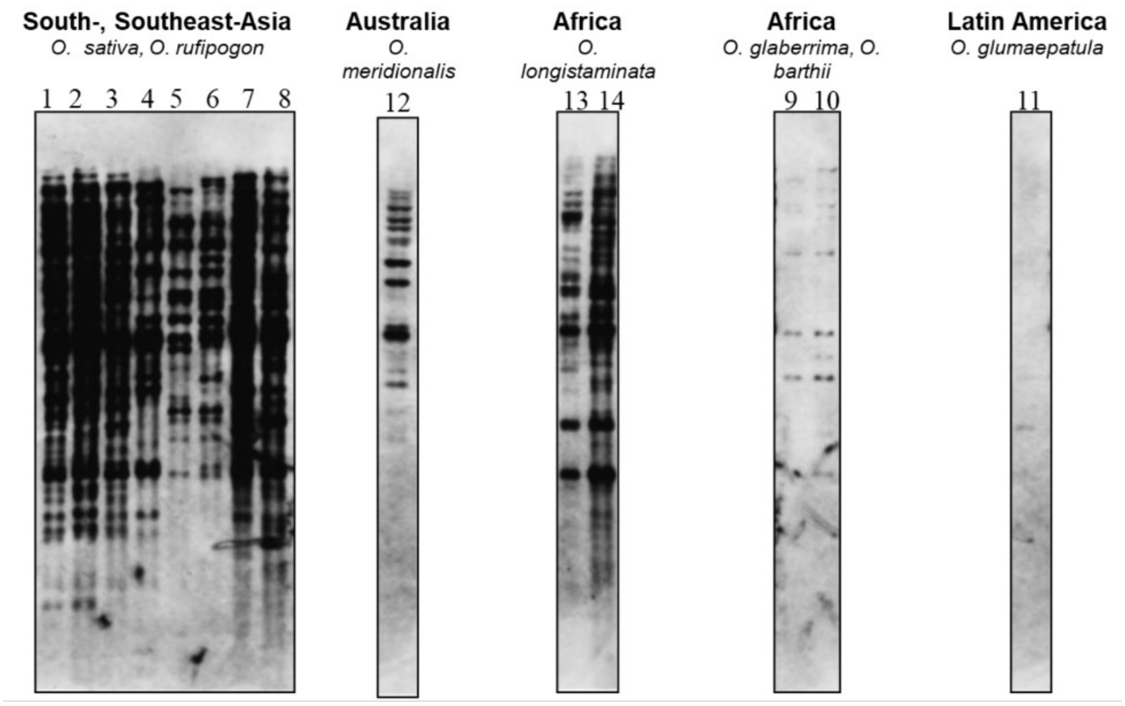

Sample	*Species*	Cultivar or accession	Remarks
1	*O. sativa*	Shimokita	Japonica from Japan
2	*O. sativa*	T65wx	Near-isogenic line of Taichung 65 with wx from Kinoshitamochi (BC12)
3	*O. sativa*	221	Javanica type from Indonesia
4	*O. sativa*	PTB10	Indica type from India
5	*O. rufipogon*	W107	Annual type from India
6	*O. rufipogon*	W120	Perennial type from India
7	*O. rufipogon*	W1717	Perennial type from China (through IRRI)
8	*O. rufipogon*	W1718	Perennial type from China (through IRRI)
9	*O. glaberrima*	W025	From Guinea
10	*O. barthii*	W1592	From Cameroon
11	*O. glumaepatula*	W1185	From Surinam
12	*O. meridionalis*	W1625	From Australia
13	*O. longistaminata*	W1034	From Nigeria
14	*O. longistaminata*	W1572	From Nigeria

Supplementary Figure 5
Evidence for introgression of chromosomal segments from *japonica* to *O. longistaminata* using eRTBVL sequences.
Southern blotting patterns (upper) are from Figure 5 of Kunii et al. (2004). Genomic DNAs from 14 *Oryza* lines probed with a 7.4-kb eRTBVL fragment. The numbers above the blots correspond to the numbered materials in the table (bottom).

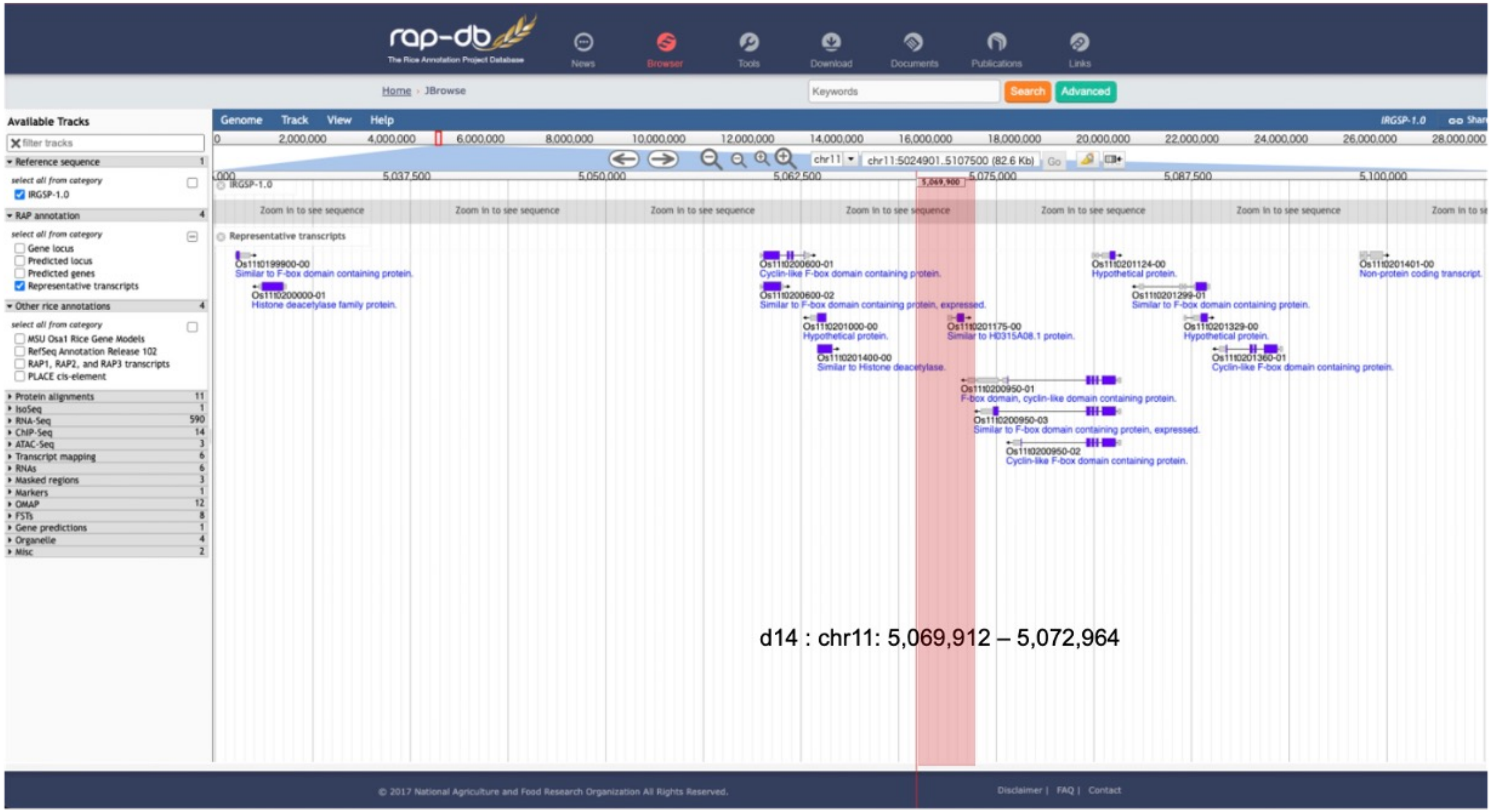

https://rapdb.dna.affrc.go.jp/jbrowse/?data=data%2Firgsp1&loc=chr11%3A5024901..5107500&tracks=IRGSP-1.0%2CRepresentative%20transcripts&highlight=

Supplementary Figure 6
Genome annotation around the d14 segment.
The screenshot was obtained from RAP-DB (https://rapdb.dna.affrc.go.jp/). The gene structure, including the exons and introns, is shown in purple, and the d14 segment region is shaded in red.
